# Unveiling the role of hypoxic macrophage-derived exosomes in driving colorectal cancer progression

**DOI:** 10.3389/fimmu.2023.1260638

**Published:** 2023-11-09

**Authors:** Jiang Jiang, Wenfang Wang, Lan Zhu, Bowen Shi, Yong Chen, Yihan Xia, Weiming Feng, Weiwu Yao, Aiguo Lu, Huan Zhang

**Affiliations:** ^1^ Department of Radiology, Ruijin Hospital, Shanghai Jiao Tong University School of Medicine, Shanghai, China; ^2^ Department of Imaging, Tongren Hospital, Shanghai Jiao Tong University School of Medicine, Shanghai, China; ^3^ Department of General Surgery, Ruijin Hospital, Shanghai Jiao Tong University School of Medicine, Shanghai, China

**Keywords:** colorectal cancer, hypoxia, macrophages, exosomes, Hsp90, Yap

## Abstract

The crosstalk between tumor cells and macrophages under hypoxic conditions has been acknowledged as a pivotal determinant in the progression of colorectal cancer (CRC). Previous research has underscored the significance of exosomes derived from hypoxic tumor cells in facilitating tumor progression through inducing the polarization of macrophages towards the M2-like phenotype. The precise influence of hypoxic macrophage-derived exosomes (HMDEs) on the progression of CRC has not yet been fully elucidated. The objective of this study was to investigate the role of HMDEs in the progression of CRC. We discovered that there was an elevated release of exosomes derived from macrophages in hypoxic conditions. Additionally, the hypoxia-induced macrophage-derived exosomes played a crucial role in promoting the progression of CRC. We have also demonstrated that HMDEs have the ability to induce cell cycle transition and inhibit cell apoptosis, thereby promoting the growth of CRC cells. Furthermore, the underlying molecular mechanisms of these effects have been identified. The overexpression of Hif-1α results in its direct interaction with distinct regions (-521− -516 bp and -401− -391 bp) of the *Hsp90* promoter during hypoxic circumstances. This binding event led to the overexpression of Hsp90 and the subsequent elevation of Hsp90 protein levels within HMDEs. Importantly, the crucial interaction between Hsp90 and Lats1 resulted in the deactivation of Lats1 and the inhibition of Yap phosphorylation. Ultimately, this series of events lead to the deactivation of the Hippo signaling pathway. Our *in vivo* and *in vitro* studies presented compelling evidence for the crucial role of hypoxic macrophage-derived exosomal Hsp90 in promoting CRC progression through the inhibition of the Hippo signaling pathway. These findings represented a significant advancement in our comprehension of the complex interplay between macrophages and CRC cells under hypoxic conditions.

## 1 Introduction

In 2020, colorectal cancer (CRC) accounted for approximately 1.93 million newly diagnosed cases worldwide, leading to around 0.94 million CRC-related deaths (1). The persistent progression of CRC remains the primary cause of cancer-related mortality globally (2). Researchers have identified the crosstalk between macrophages and CRC cells as a crucial factor contributing to CRC progression, especially in the context of hypoxic conditions (3, 4). Hypoxia refers to low oxygen levels in the tumor microenvironment and promotes the polarization of tumor-associated macrophages (TAMs) towards the M2 phenotype. This process involves the activation of several signaling pathways, including Akt-mTOR, TGF-β, and ERK pathways (5, 6). Under hypoxic conditions, M2 macrophages have been observed to promote tumor progression in lung cancer and breast cancer by increasing the secretion of exosomes (5, 7, 8). Exosomes are small vesicles released by cells containing various molecules, such as proteins, nucleic acids, and lipids. Tumor-derived exosomes, extensively studied, play a role in promoting recurrence and metastasis in breast cancer, lung cancer, and CRC (7, 9, 10). Despite this knowledge, the specific effects of hypoxia on the composition of macrophage-derived exosomes and their implications in regulating CRC progression have not yet been fully understood. Further research is required to investigate how hypoxia alters the content of macrophage-derived exosomes and how these changes contribute to the regulation of CRC progression.

To understand the underlying mechanisms, scholars direct their attention toward the Hippo signaling pathway, which is recognized for its role in governing cell proliferation and apoptosis. This pathway has been implicated in a range of cancers, including CRC (11–13). Dysregulation of this pathway has been implicated in tumorigenesis and the progression of various cancers, including CRC (14). The downstream effector, Yes-associated protein (YAP), translocates to the nucleus upon dephosphorylation. It forms complexes with transcription factors that promote target gene expression (15, 16). LATS1/2, when activated, inhibits YAP nuclear translocation by phosphorylation. Previous studies have reported that YAP nuclear translocation contributes to M2 macrophage-derived exosome-mediated lung adenocarcinoma progression (15). However, the involvement of the Hippo signaling pathway in macrophage-derived exosome-mediated CRC progression, especially under hypoxic conditions, has yet to be explored.

In this study, our objective is to investigate the role of hypoxic macrophage-derived exosomes (HMDEs) in the progression of CRC. By conducting an investigation into the modifications occurring in exosomes derived from macrophages and their impact on CRC via the Hippo signaling pathway, our aim is to provide novel perspectives on potential therapeutic strategies for the more efficient management of CRC.

## 2 Materials and methods

### 2.1 Recruitment of colorectal cancer (CRC) patients and healthy volunteers

We obtained informed consent and approval from both CRC patients and healthy volunteers for the collection of all serum samples used in this study, ensuring strict adherence to ethical regulations. We enrolled a total of twenty CRC patients and ten healthy volunteers in the study for the purpose of analysis and research.

### 2.2 Cell culture and compound

We acquired the murine CT-26 cell line and murine RAW264.7 cell line from the American Type Culture Collection (ATCC). Both cell lines were cultured at 37°C in a 5% CO_2_ atmosphere in RPMI 1640 medium supplemented with 10% fetal bovine serum (Gibco, Thermo Fisher) and 1% penicillin/streptomycin (Yeasen, Shanghai, China). To generate bone marrow-derived macrophages (BMDMs), we employed male C57BL/6 J mice aged 6−8 weeks. We isolated BMDMs from the femurs and tibias of the mice and subsequently cultured them in high-glucose DMEM supplemented with 10% fetal bovine serum (Gibco, Thermo Fisher), 1% penicillin/streptomycin, and 50 ng/ml granulocyte-macrophage colony-stimulating factor (GM-CSF) for a duration of seven days to facilitate their differentiation into mature macrophages (17). For the purpose of creating hypoxic conditions, both RAW264.7 cells and BMDMs were subjected to a hypoxic chamber with an oxygen concentration of 1%, while a normoxic atmosphere with 21% oxygen was used for normoxic conditions. The specific duration of exposure to the respective oxygen levels was as indicated for the individual experiments.

### 2.3 Macrophage-derived supernatant collection

We replaced the culture medium of RAW264.7 cells or BMDMs with fresh RPMI 1640 or DMEM supplemented with 10% exosome-depleted FBS, followed by cultivation under either normoxic conditions (21% O_2_) or hypoxic conditions (1% O_2_) for a duration of 12 hours. After this 12-hour period, we collected the supernatants containing secreted factors from the macrophages, referred to as cell supernatants. To purify the cell supernatants, we subjected them to sequential centrifugation steps: 300 × g for 10 minutes, followed by 1500 × g for 30 minutes, and finally at 10,000 × g for 60 minutes, with the aim of eliminating any residual cells and debris. Subsequently, we cocultured CT-26 cells with these macrophage-derived supernatants for approximately 48 hours. We observed and documented the morphological alterations of the CT-26 cells using inverted microscopy. Additionally, we assessed the viability of the CT-26 cells utilizing a CCK-8 assay, measuring the cells’ metabolic activity and providing insights into cell proliferation and viability.

### 2.4 Exosome extraction

We replaced the culture medium of RAW264.7 cells and BMDMs with fresh medium and subsequently exposed the cells to either normoxic conditions (21% O_2_) or hypoxic conditions (1% O_2_) for the designated time frame. Following the incubation period, we collected the supernatants containing exosomes secreted by the macrophages for subsequent analysis. To extract exosomes from the supernatants, we utilized two different methods. For the macrophage-derived supernatant, we employed an Exosome Isolation Kit (EZBioscience, USA) in accordance with the manufacturer’s instructions. This kit enabled the efficient retrieval of exosomes from the collected supernatant. Regarding serum samples, we isolated serum exosomes using a Hieff^®^ Quick exosome isolation kit (Yeasen, China) as per the manufacturer’s instructions. Subsequently, we resuspended the isolated exosomes in PBS, quantified them, and prepared them for further analysis.

### 2.5 Exosome quantification

To determine the size and quantity of the isolated particles, we analyzed vesicle suspensions with concentrations ranging from 1 × 10^7/mL to 1 × 10^9/mL using a ZetaView PMX 110 instrument (Particle Metrix, Meerbusch, Germany). The instrument is equipped with a 405 nm laser and is capable of recording a 1-minute video at a frame rate of 30 frames per second. We analyzed the captured video using Nanoparticle Tracking Analysis (NTA) software (ZetaView 8.02.28) to measure particle movement and provided insights into the size and quantity of particles in the vesicle suspensions. For further exosome characterization, we performed an additional step. We placed the exosome solution on a copper mesh and allowed it to incubate at room temperature for 10 minutes. Following incubation, we washed the copper mesh with sterile distilled water and treated the exosomes with a uranyl-oxalate solution for 1 minute to enhance contrast. Subsequently, we dried the samples for 2 minutes under incandescent light. We then employed a transmission electron microscope (H-7650, Hitachi Ltd., Tokyo, Japan) to observe and photograph the copper meshes, enabling visualization of the exosomes and examination of their morphology and structure.

### 2.6 CCK‐8 assay

For the cell viability assay, we plated 5 × 10^3 CT-26 cells in each well of a 96-well plate and permitted them to incubate for 24 hours. Subsequent to this initial incubation, we subjected the cells to coincubation with exosomes for an additional 24 to 96 hours. To evaluate cell viability, we supplemented each well with 10 µL of CCK‐8 reagent (Meilun, China). Subsequently, we returned the 96-well plates to the incubator and allowed them to incubate for an additional 2 hours. Following this incubation period, we measured the optical density (OD) value at 450 nm using a microplate reader.

### 2.7 Proteomics analysis

We utilized a total of 1 μg of protein isolated from exosomes under both normoxic and hypoxic conditions for modern mass sequencing analysis. To disrupt the proteins, we employed agitation with a homogenizer (Fastprep-24^®^, MP Biomedical) and subsequently boiled them for 5 minutes. The samples underwent additional ultrasonication and another 5-minute boiling. Centrifugation at 14,000 rpm for 15 minutes was then performed to eliminate any undissolved debris. Following this step, we collected the supernatants and quantified them using a BCA Protein Assay Kit (Bio-Rad, USA). To perform protein digestion (250 μg for each sample), we followed the FASP procedure. This involved eliminating detergents, DTT, and other low-molecular-weight components using 200 μL of UA buffer (8 M Urea, 150 mM Tris-HCl pH 8.0) through repeated ultrafiltration facilitated by centrifugation. Subsequently, 100 μL of 0.05 M iodoacetamide in UA buffer was added to block reduced cysteine residues, and the samples were incubated in the dark for 20 minutes. We washed the filters with 100 μL of UA buffer three times, followed by 100 μL of 25 mM NH_4_HCO_3_ twice. The protein suspensions were then digested using 3 μg of trypsin (Promega) in 40 μL of 25 mM NH4HCO3 overnight at 37°C, and the resulting peptides were collected as a filtrate. The peptide content of each sample was estimated using UV light spectral density at 280 nm, with an extinction coefficient of 1.1 of 0.1% (g/L) solution calculated based on the tryptophan and tyrosine frequency in vertebrate proteins. Subsequently, we desalted the peptides of each sample using C18 Cartridges (Empore™ SPE Cartridges C18) and concentrated them through vacuum centrifugation before reconstituting them in 40 µL of 0.1% (v/v) trifluoroacetic acid. MS experiments were conducted using a Q Exactive mass spectrometer coupled with Easy nLC (Proxeon Biosystems, now Thermo Fisher Scientific). We loaded five micrograms of peptide onto a C18-reversed-phase column (Thermo Scientific Easy Column, 10 cm long, 75 μm inner diameter, 3 μm resin) in buffer A (2% acetonitrile and 0.1% formic acid) and separated them with a linear gradient of buffer B (80% acetonitrile and 0.1% formic acid) at a flow rate of 250 nL/min controlled by IntelliFlow technology over 120 minutes. The described service was provided by Shanghai Personal Biotechnology Co., Ltd. (Shanghai, China). Detailed information can be found in **Supplementary 1**.

### 2.8 High-throughput sequencing

We extracted total RNA from CT-26 cells that had been cocultured with exosomes under both normoxic and hypoxic conditions using TRIzol reagent (Thermo Scientific). For each sample, we generated and analyzed three replicates. To construct an RNA-seq transcriptome library, we prepared one milligram of RNA following the TruSeqTM RNA Sample Preparation Kit from Illumina (San Diego, CA). Firstly, we isolated messenger RNA (mRNA) through the polyA selection method using oligo (dT) beads, followed by fragmentation with fragmentation buffer. Subsequently, we synthesized double-stranded cDNA using a SuperScript double-stranded cDNA synthesis kit (Invitrogen, CA) with random hexamer primers (Illumina). The synthesized cDNA underwent end repair, phosphorylation, and ‘A’ base addition as per Illumina’s library construction protocol. We then performed size selection on the libraries to obtain cDNA target fragments of 300 bp length using 2% Low Range Ultra Agarose. This was followed by PCR amplification using Phusion DNA polymerase (NEB) for 15 PCR cycles. After quantification with TBS380, we sequenced the paired-end RNA-seq sequencing library using a NovaSeq 6000 sequencer (2 × 150 bp read length). The obtained reads were aligned to the mm10 (Mus) reference genome. For the subsequent analysis, we conducted GO functional enrichment and KEGG pathway analyses on the Majorbio Cloud Platform (www.majorbio.com). Detailed information can be found in **Supplementary 2**.

### 2.9 ELISA

We conducted ELISA according to previously established procedures. Subsequent to collecting serum samples, we quantified the levels of HSP90 protein in exosomes using ELISA kits (Bio TNT, Shanghai, China) as per the manufacturer’s instructions.

### 2.10 Immunofluorescence staining assays (IF) and confocal microscopy

We conducted immunofluorescence staining assays (IF) following previously established methods (18).

### 2.11 Apoptosis analysis and cell cycle analysis by flow cytometry

We assessed apoptosis using flow cytometry, and the detailed protocol was documented in our previous study (18).

### 2.12 RNA interference

We designed and synthesized *Hsp90* siRNA (Genomeditech, China), *Hif-1α* siRNA, and negative control sequences through Genomeditech (Tsingke Biotechnology Co., Ltd, China). The siRNA molecules were transfected into macrophages at a final concentration of 100 nmol/L using ViaFect™ (Promega Corporation, China), following the manufacturer’s instructions. The cells were collected for subsequent assays at 24, 48, and 72 hours after transfection.

The *Hsp90* siRNA#1 sense sequence was 5’-GCUGAUACCUGAGUACCUCAAtt-3’, and the antisense sequence was 5’-UUGAGGUACUCAGGUAUCAGCtt-3’;

the *Hsp90* siRNA#2 sense sequence was 5’-CCAGCUCAUGUCCCUCAUCAUCAUtt-3’, and the antisense sequence was 5’- AUGAUGAGGGACAUGAGCUGGtt-3’;

and the *Hsp90* siRNA#3 sense sequence was 5’- CCGCAAGAACAUCGUCAAGAAtt-3’, and the antisense sequence was 5’-UUCUUGACGAUGUUCUUGCGGtt -3’.

The *Hif1-α* siRNA#1 sense sequence was 5’- GCUUUGAUGUGGAUAGCGAtt-3’, and the antisense sequence was 5’- UCGCUAUCCACAUCAAAGCtt-3’;

the *Hif1-α* siRNA#2 sense sequence was 5’-GGUGACUGUGCACCUACUAtt-3’, and the antisense sequence was 5’- UAGUAGGUGCACAGUCACCtt-3’;

and the *Hif1-α* siRNA#3 sense sequence was 5’-GACUCAGCUGUUCACCAAAtt-3’, and the antisense was 5’- UUUGGUGAACAGCUGAGUCtt-3’.

### 2.13 RNA extraction and quantitative polymerase chain reaction (qPCR)

We isolated total RNA from RAW264.7 cells, BMDMs, and CT-26 cells using an EZ-press RNA Purification Kit (EZBioscience, China, B0004D) following the manufacturer’s instructions. We synthesized cDNA using a reverse transcription kit from Invitrogen. For quantitative PCR (qPCR), we utilized TaqMan^®^ Gene Expression Assays from Thermo Fisher Scientific. As the internal control, we employed actin. The PCR primers utilized are provided in **Table 1**.

**Table 1:** Quantitative real-time polymerase chain reaction assay (Q-PCR) primer.

gene name	squence
hif-1a F	GATGACGGCGACATGGTTTAC
hif-1a R	CTCACTGGGCCATTTCTGTGT
yap F	ACCCTCGTTTTGCCATGAAC
yap R	TTGTTTCAACCGCAGTCTCTC
Taz F	ATGGGCCTAGTTGGCACCTA
Taz R	ATCCCTTTCTGGTAGACACCAT
Mst F	GCAAAACGCAACACTGTAATAGG
Mst R	AGCCCTCATCGGATGTATATCAG
Tead-2 F	TCTGACCTACCAGGGTACGAG
Tead-2 R	GGTTCCACAAACGCTGAGAA
Actin F	AGTTGGAAAGATGGTCTTGGTTT
Actin R	GCAGTTCCGACGATGTCTTCA
Lats-2 F	GGACCCCAGGAATGAGCAG
Lats-2 R	CCCTCGTAGTTTGCACCACC
Lats-1 F	TCCGGCATTGCAGCATTTG
Lats-1 R	AGGGGAGATTCGGGAGATTAC
14-3-3-F	GTTCGACCTCCTGAGAAAAGG
14-3-3-R	GACACCAAAGTCGGCGATCTT
hsp90 F	TCAAACAAGGAGATTTTCCTCCG
hsp90 R	GCTGTCCAACTTAGAAGGGTC
Ctgf F	AGCTGACCTGGAGGAAAACA
Ctgf R	GACAGGCTTGGCGATTTTAG
Cyr61 F	GCTCAGTCAGAAGGCAGACC
Cyr61 R	GTTCTTGGGGACACAGAGGA

### 2.14 Western blotting (WB)

We carried out Western blotting (WB) using established methods. In brief, cells or exosomes were lysed in RIPA lysis buffer supplemented with complete protease and phosphatase inhibitor cocktails. Subsequently, 25−30 µg of protein sample was loaded onto 4−20% Bis-Tris-PAGE gels (Tanon, China) and then transferred onto 0.22 µm polyvinylidene difluoride membranes (Millipore). The membranes were visualized using chemiluminescent (ECL) Western Blotting Substrate (NCM Biotech, Suzhou, China) and a Tanon 5200 system (Tanon). For primary antibodies, we employed anti-CD9, anti-CD81, anti-TSG101, anti-HSP70, anti-ALIX, anti-CD206, anti-arginase-1, and anti-flotillin-1 (1:1000; Cell Signaling Technology); anti-HIF-1α, anti-HSP90, and anti-ACTIN (1:1,000; Proteintech); as well as anti-LATS1, anti-p-LATS1, anti-YAP, anti-activated-YAP, and anti-p-YAP (1:1,000; Cell Signaling Technology). For secondary antibodies, we utilized anti-mouse IgG (1:5000, Cat No. SA00001-1, Proteintech) and anti-rabbit IgG (1:5000, Cat No. SA00001-2, Proteintech).

### 2.15 Xenograft tumor model

We randomly allocated ten 4-week-old male BALB/c nude mice (Beijing Vital River Laboratory Animal Technology Co., Ltd, China) into two groups, with 5 mice per group. All the animal experiments, with approval from the Institutional Animal Care and Use Committee, were conducted in adherence to the institution’s guidelines and principles for animal research. Subsequently, we subcutaneously injected a total of 5 × 10^4 CT-26 cells into the mice. Upon the formation of subcutaneous nodules, we administered exosomes to the mice through tail vein injections. Following a period of 3−4 weeks, all the mice were humanely sacrificed under general anesthesia. We removed, weighed, processed, and embedded the tumor masses in paraffin for further investigation.

### 2.16 Chromatin immunoprecipitation (ChIP) assay

We conducted Chromatin immunoprecipitation (ChIP) assays following established procedures. To perform these assays, we utilized a total of 2 × 10^7 RAW264.7 cells. Formaldehyde fixation (Sigma, Thermo Fisher) was conducted for a duration of 10 minutes. The ChIP procedure was executed as per the manufacturer’s instructions (SimpleChIP, CAT# CST9002, CST). During this process, chromatin was subjected to immunoprecipitation using anti-HIF1-α (CAT# ab272660, CST, 10 mg/mL) and IgG antibodies (CAT# 3900S, CST, 10 mg/mL), which had been preincubated with protein A/G magnetic beads. After three rounds of washing, the beads were used to elute, de-crosslink, and purify the DNA. To facilitate the amplification and visualization of the PCR products, we employed primers designed using target gene promoter regions as templates (**Supplementary 1**). The resulting PCR products were separated and observed using a 2.5% agarose gel. For further quantification, purified DNA was subjected to genomic qPCR using serial dilutions of the input as a standard curve, with triplicate analyses performed. The outcomes were represented as the percent enrichment of bound DNA compared to each corresponding input. The specific primers used for both PCR and qPCR are provided in **Table 2**.

**Table 2:** The information of primers used in the ChIP assay.

name	primer
P1F	TCAGGTCCGCCTCTGAAAAC
P1R	ATCTTGTGTCCGCGGGAAAT
P2F	CCAAAAGAAAGGTGACGGCG
P2R	GGGAAACGGTAGCCAAGACA
P3F	CGCCCAAAAGAAAGGTGACG
P3R	AACCACTTCCGGCTTCTAGC
P4F	TGTCTTGGCTACCGTTTCCC
P4R	ATGAACGTAGGCGCATAGCA
P5F	CGCTCTCTGGCTGATTCACA
P5R	AGGGTCCCTAGAGCCTTCAG
P6F	AGAGGACAGGAAATGCGAGC
P6R	CAGTTTTCAGAGGCGGACCT
P7F	CAGGTCCGCCTCTGAAAACT
P7R	CGCGGGAAATGTCTCTGAGT
P8F	CTGAGTGACGCGCAAGAAAG
P8R	GCACACAGTTTTCAGAGGCG
P9F	ATTTCCCGCGGACACAAGAT
P9R	GGAATGCGTTTGGATGGTGG
P10F	GCTATCTTTCGGAAGGCGGA
P10R	TGTGAATCAGCCAGAGAGCG
P11F	GTAGCGCTGAAAGGACTCGT
P11R	ACTTCCGGCTTCTAGCGAAC
P12F	ACGTAGCGCTGAAAGGACTC
P12R	CACTTCCGGCTTCTAGCGAA
P13F	CCACCATCCAAACGCATTCC
P13R	GTCCGTGACTAGGGGAAACG
P14F	GCTAGAAGCCGGAAGTGGTT
P14R	CCTCCAATCAGATGGCTCCG
P15F	CACCATCCAAACGCATTCCG
P15R	ACGAGTCCTTTCAGCGCTAC
P16F	TGGGTCCTTGTTTCCTTCGG
P16R	GGATTCTGGGCTGGTGGTAG
P17F	GGATGTGTTAGAGGCAGGCA
P17R	TCCGCCTTCCGAAAGATAGC
P18F	TCACGGACTAAACGTTCGCT
P18R	TTGGTCCCTGAGGAAAACCG
P19F	TTTCCCGCGGACACAAGATT
P19R	CGGAATGCGTTTGGATGGTG
P20F	GTTCGCTAGAAGCCGGAAGT
P20R	GCGGCTTTCCTCCAATCAGA

### 2.17 Luciferase reporter assay

A luciferase reporter assay was performed as previously described (18). Briefly, the 2.1-kb *Hsp90* promoter (WT and Mut) was cloned into the pGL3-Basic luciferase reporter vector. The activity of the *Hsp90* promoters was normalized by co-transfection with the Renilla luciferase reporter plasmid, which was a gift from Fudan University. Firefly and Renilla luciferase activities were measured 48 h after transfection using a dual-luciferase reporter assay system (Promega).

### 2.18 Statistical analysis

The experimental results were obtained from three or five independent experiments, and the data are shown as the mean ± standard deviation (SD). The differences between two groups were analyzed by Student’s t test, while the differences between more than two groups were analyzed by one-way or two-way analysis of variance (ANOVA). *p* < 0.05 is considered to be the criterion for statistical significance. All the statistical analyses were performed with GraphPad Prism.

## 3 Results

### 3.1 Supernatant derived from macrophages under hypoxic conditions promotes the proliferation of CT-26 cells

**Figure 1:**
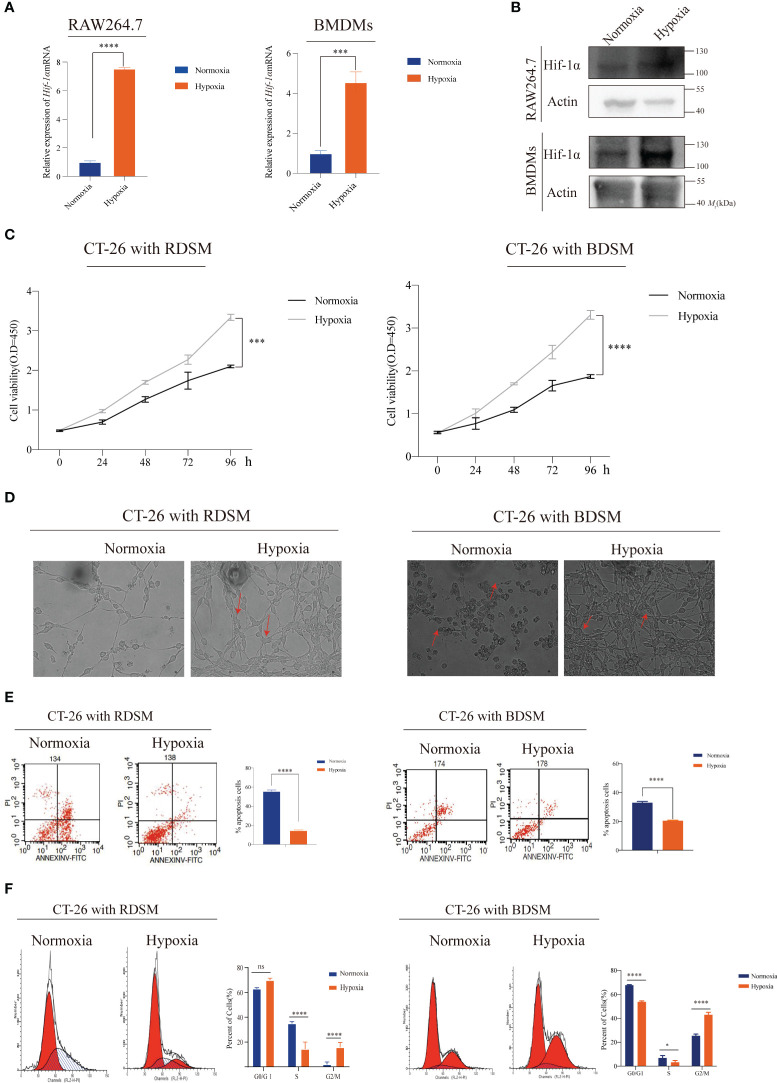
Supernatant derived from macrophages under hypoxic conditions promotes the proliferation of CT-26 cells **(A, B)** Increased mRNA and protein levels of Hif-1α were observed in RAW264.7 cells (***P < 0.001, ****P < 0.0001); **(C)** A CCK-8 assay was performed to confirm CT-26 cell proliferation after coculture with supernatant medium from macrophages under hypoxic conditions (***P < 0.001, ****P < 0.0001); (RDSM: RAW264.7 cell-derived supernatant medium; BDSM: BMDM-derived supernatant) **(D)** Co-culturing CT-26 cells with hypoxia-derived supernatant from RAW264.7 cells or BMDMs resulted in a healthier and more elongated cellular morphology. This implies a potentially favourable influence of the hypoxic environment on cell morphology; **(E)** Flow cytometry was employed for cell cycle analysis to validate the role of macrophages in modulating the cell division cycle under hypoxic conditions (****P < 0.0001) **(F)** Flow cytometry was utilized to perform apoptosis analysis, aiming to investigate the contribution of macrophages to cell apoptosis under hypoxic conditions. (ns, notsignificant, *P < 0.05, ****P < 0.0001).

Hypoxia is a well-established factor in reshaping the tumor microenvironment and influencing tumor progression (8, 19). In response to hypoxia, hypoxia-inducible factors (HIFs) act as critical transcription factors, with HIF-1α being a key player among the isoforms involved in the cellular response to low oxygen conditions (20, 21). In our study, we conducted qPCR and WB assays to confirm the activation of HIF-1α after subjecting these cell lines to hypoxic conditions (1% O_2_) for 10−12 hours (**Figures 1A, B**, and **S1A, S1B**). Following this, we delved into the impact of hypoxic macrophages on tumor cell growth. Supernatants derived from hypoxic RAW264.7 cells or BMDMs were utilized for co-culture with CT-26 cells. The results revealed a significant enhancement in the proliferation of CT-26 cells when exposed to these supernatants from hypoxic macrophages, as compared to those treated with supernatants from normoxic macrophages (**Figure 1C**). Notably, CT-26 cells co-cultured with supernatants from hypoxic macrophages exhibited a more robust cell morphology compared to those co-cultured with supernatants from normoxic macrophages (**Figure 1D**). This observation suggests a potential role for macrophages in promoting tumor growth under hypoxic conditions.

To gain deeper insights into the underlying mechanisms, we further investigated the effects of hypoxic macrophages on cell cycle progression and apoptosis through flow cytometry assays. The results consistently demonstrated that hypoxic macrophages facilitated cell cycle transition and concurrently inhibited cell apoptosis (**Figures 1E, F**). In conclusion, our findings provide compelling evidence that supernatants from hypoxic RAW264.7 cells or BMDMs can promote CT-26 cell proliferation by inducing cell cycle transition and suppressing apoptosis.

### 3.2 Hypoxia promotes exosomes secretion of macrophages

**Figure 2:**
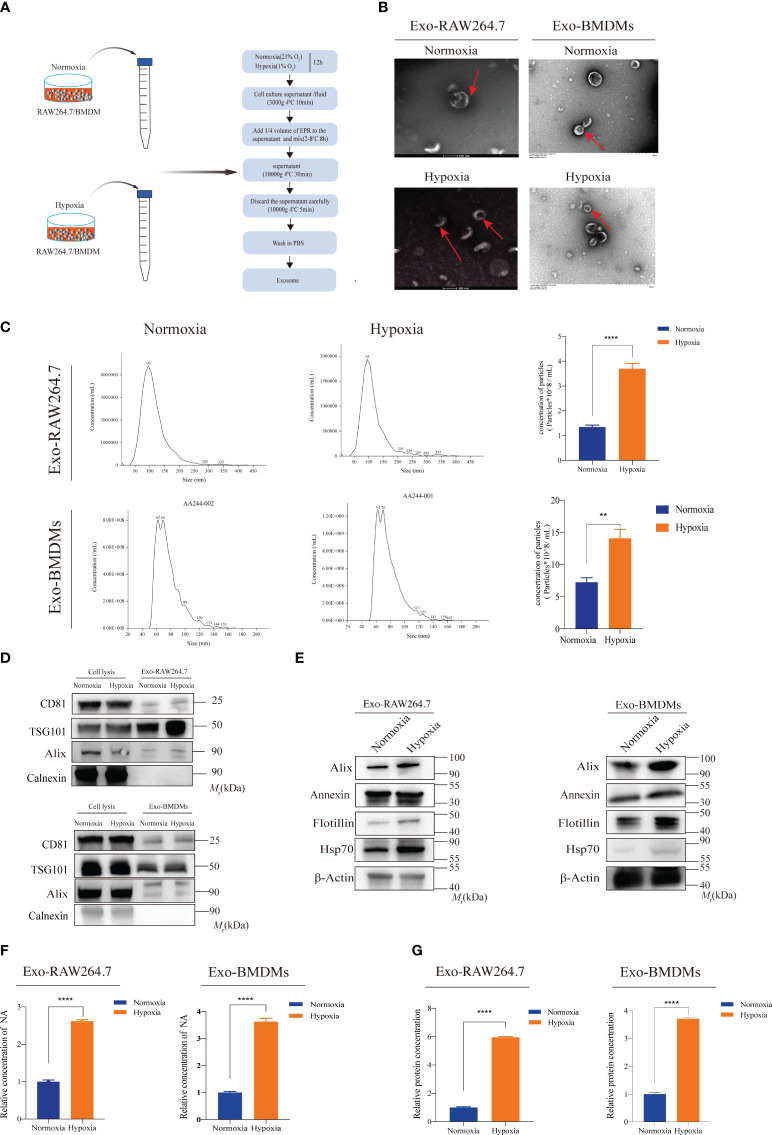
Hypoxia promotes exosomes secretion of macrophages **(A)** The schematic diagram illustrates the exosome isolation process from supernatant medium derived from normoxic or hypoxic macrophages. **(B)** TEM images depict the diameter and morphology of exosome vesicles isolated from the conditioned medium of macrophages under normoxic and hypoxic conditions; **(C)** NTAwas employed to assess the size of exosomes derived from normoxic and hypoxic macrophages, as well as to quantify the exosome concentration in the conditioned medium (^∗∗^P < 0.01, ****P < 0.0001); **(D)** WB of exosome markers CD81, TSG101, Alix, and Calnexin in the cell lysis or extracted exosomes from RAW264.7 or BMDM cells treated without or with hypoxia; **(E)** WB analysis for exosome protein markers, such as Hsp70, Flotillin-1, Annexin, and Alix; **(F, G)** Hypoxia increased the concentrations of nucleic acids and proteins in exosomes (****P < 0.0001).

In previous studies, it has been demonstrated that hypoxia can enhance the secretion of exosomes derived from tumors, which in turn contributes to tumor progression (9). However, it still remains unclear whether hypoxia indeed influences the release of exosomes from macrophages. Therefore, we conducted a comprehensive investigation into the impact of hypoxia on macrophage exosome secretion. RAW264.7 cells and BMDMs were exposed to both normoxic (21% O_2_) and hypoxic (1% O_2_) conditions for a period of 12 hours. Subsequently, exosomes were isolated, purified, and quantified (**Figure 2A**). TEM confirmed the presence of vesicles exhibiting the characteristic morphology typical of exosomes. Notably, there were no observable differences in the morphology of exosomes between the normoxic and hypoxic groups (**Figure 2B**). The results obtained from NTA demonstrated that the average diameter of the isolated vesicles was approximately 100 nm, which aligns with the typical size range of exosomes (**Figure 2C**). Furthermore, NTA analysis revealed a higher concentration of exosomes derived from both RAW264.7 and BMDM cells after exposure to 1% O_2_ for 12 hours, as compared to normoxic conditions (**Figure 2C**). Furthermore, WB analysis yielded compelling evidence of the presence of exosome markers (CD81, TSG101, ALIX, and Calnexin) within exosomes derived from both RAW264.7 and BMDM cells, regardless of the oxygen conditions (**Figure 2D**). In accordance with the findings from the NTA, the WB assay examined proteins obtained from exosomes and validated increased levels of exosomal proteins such as Alix, Annexin, Flotillin-1, and Hsp70 in the hypoxic group (**Figure 2E**, **S2A**, and **S2B**). Additionally, we observed a significant increase in protein and nucleic acid levels within exosomes under hypoxic conditions. This elevation was consistently demonstrated through exosome labeling, WB analysis, and Nanodrop analysis (**Figures 2F, G**). In summary, these comprehensive findings unequivocally confirm that hypoxia enhances the secretion of exosomes by macrophages.

### 3.3 Hypoxic macrophages enhance the proliferation of CT-26 cells by increasing exosome release

**Figure 3:**
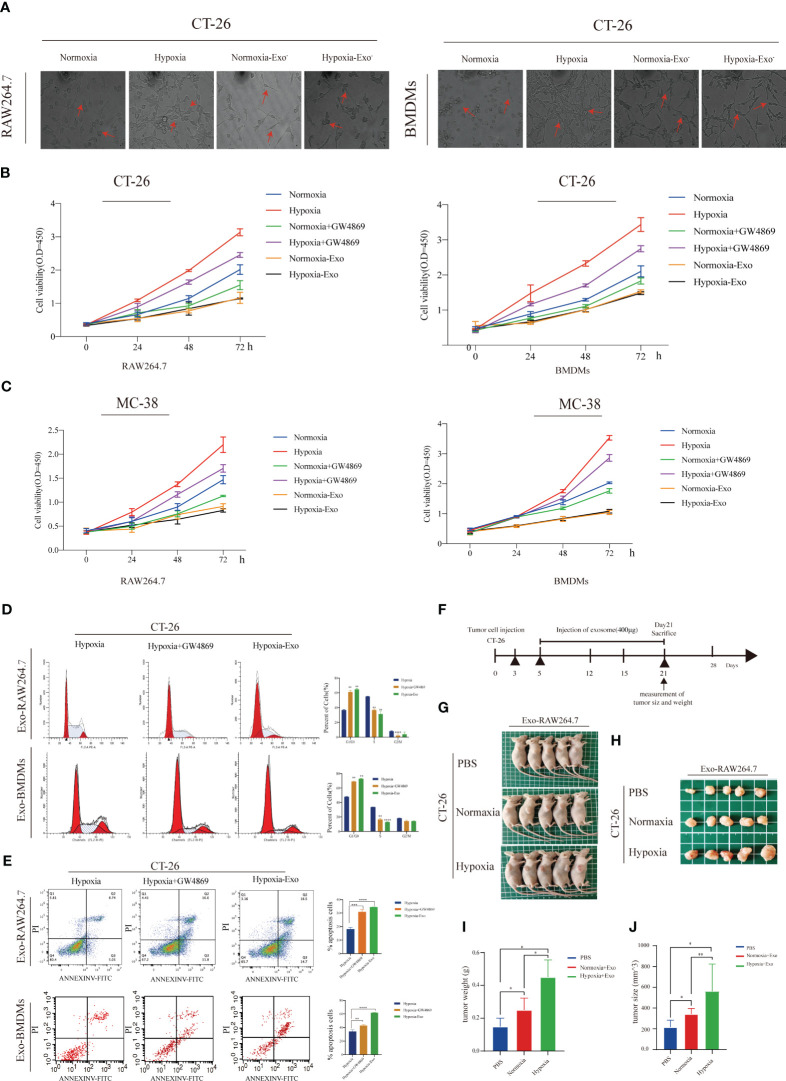
Hypoxic macrophage-derived exosomes (HMDEs) play a role in promoting tumor growth by inhibiting the Hippo signaling pathway **(A)** The morphology analysis revealed that CT-26 cells were cocultured with supernatant medium under both hypoxic and normoxic conditions, with or without prior exosome removal using ultracentrifugation; **(B, C)** The CCK-8 assay demonstrated that HMDEs played a role in promoting the growth of both CT-26 and MC-38 cells; **(D, E)** GW4869 was applied to confirm the role of HMDEs in regulating tumor cell-cycle transition and cell apoptosis; (^∗∗^P < 0.01, ^***^P < 0.001, ^****^P < 0.0001) **(F)** Schematic illustration showing the process of establishing a xenograft tumor model in BALB/c nude mice (400μg exosome per mouse); **(G, H)** Representative images of tumor nodes in BALB/c nude mice; **(I, J)** Tumor weight and tumor size were measured. (*P < 0.05, **P < 0.01).

Building on the aforementioned discoveries, we hypothesized that HMDEs in the supernatants might play a crucial role in regulating the proliferation of CT-26 cells. To further investigate this hypothesis, we conducted a series of experiments to evaluate the impact of HMDEs on the growth of CT-26 cells. First, we employed gradient centrifugation to effectively remove exosomes from macrophage supernatants. Subsequently, we co-cultured CT-26 cells with these exosome-depleted supernatants. Intriguingly, CT-26 cells co-cultured with supernatants generated under hypoxic conditions displayed a healthier morphology and exhibited an increase in cell number compared to those co-cultured with supernatants generated under normoxic conditions or supernatants depleted of exosomes. Notably, no significant differences were observed in cell morphology and cell number between the groups treated with exosome-depleted supernatants (**Figure 3A**). Next, we examined the impact of hypoxic macrophages on the growth of CRC cell lines. To inhibit the release of exosomes from macrophages, we employed GW4869, a blocker of exosome secretion. Our subsequent WB experiments confirmed the inhibitory effect of GW4869 on macrophage exosome secretion (**Figure S3A**). Subsequent CCK-8 assays validated the promoting effect of HMDEs on CT-26 cell proliferation (**Figures 3B, C**). Consistently, our experiments, which relied on varying exosome dosages, also confirmed the enhanced viability of CT-26 cells and MC-38 cells with higher exosome doses (**Figure S4A**). Furthermore, treatment of HMDEs with GW4869 resulted in similar changes in cell cycle transition and prevention of cell apoptosis, consistent with the previous findings (**Figures 3D, E**, **S5A** and **S5B**). To further substantiate the role of HMDEs in tumor growth *in vivo*, we injected CT-26 cells subcutaneously into nude mice, followed by injections of either PBS or HMDEs (400 μg of exosomes per mouse) (**Figure 3F**). Measurement of tumor volumes demonstrated that the group injected with HMDEs exhibited larger tumor volumes compared to the groups injected with PBS or the normoxic group (**Figures 3G, H**). Additionally, the tumor weight and volume were significantly higher in the HMDE-injected group compared to the PBS and normoxic groups (**Figures 3I, J**). Collectively, these results conclusively establish the essential role of HMDEs in promoting tumor growth under hypoxic conditions, both *in vitro* and *in vivo*.

### 3.4 Hypoxic macrophage-derived exosomes (HMDEs) play a role in promoting tumor growth by inhibiting the Hippo signaling pathway

**Figure 4:**
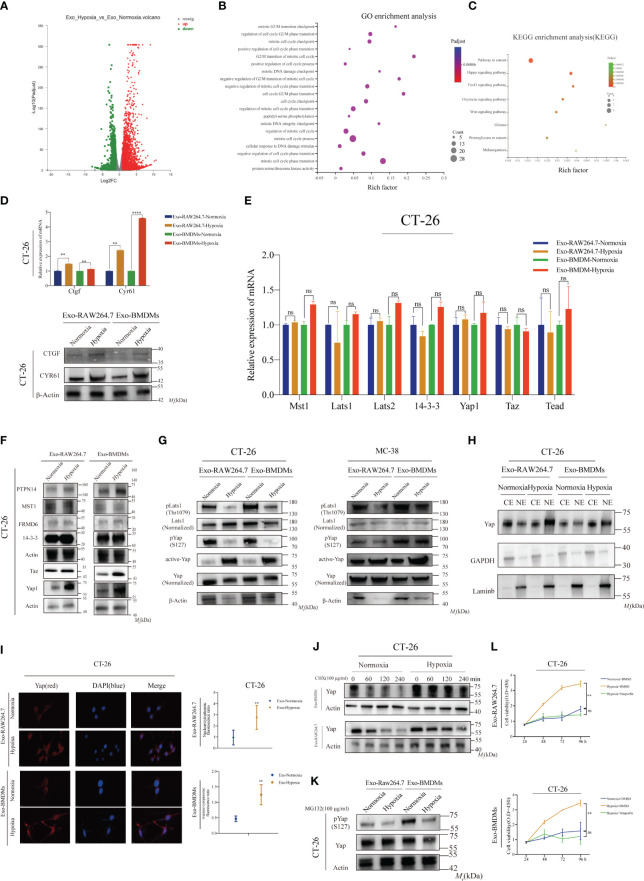
Hypoxic macrophage-derived exosomes (HMDEs) facilitate tumor growth by suppressing the Hippo signaling pathway **(A)** The differential gene expression profile of CT-26 cells comparing the NMDEs and HMDEs groups; **(B)** GO analysis of the differentially expressed genes revealed that HMDEs primarily influence cell growth by modulating genes associated with the cell cycle; **(C)** KEGG analysis showed that HMDEs contribute to tumor growth by activating the Hippo signaling pathway; **(D)**To confirm the expression of the Ctgf and Cyr61 genes at both the transcriptional and translational levels in CT-26 and MC-38 cells mediated by HMDEs; (**P < 0.01, ****P < 0.0001) **(E)** To examine the expression of the core components of the Hippo signaling pathway in HMDEs-mediated CT-26 cells, *Mst1, Lats1, Lats 2, 14-3-3, Yap1*, *Taz*, and *Tead*; (ns, not significant) **(F)** WB analysis indicated that HMDEs might regulate the Hippo signaling pathway at the translocational level; **(G)** We observed increased Yap expression and decreased Yap phosphorylation at S127 in CT-26 and MC-38 cells mediated by HMDEs. Additionally, phosphorylation of Lats1 in the hydrophobic motif (Thr1079 for Lats1) also increased; **(H, I)** Increasing Yap nuclear accumulation was observed in CT-26 cells mediated by HMDEs; (**P < 0.01) **(J)** Yap protein levels decreased more slowly in CT-26 cells mediated by HMDEs than those in the CT-26 cells mediated by NMDEs group after treatment with CHX (100 µg/mL); **(K)** CT-26 cells mediated by HMDEs regulated Yap activation by regulating the degradation of Yap via the proteasome pathway; **(L)** CCK-8 assays were performed to validate the impact of Hippo signaling pathway activation on the regulation of cell growth (ns, not significant, **P < 0.01).

To investigate the mechanism underlying HMDEs-induced CT-26 cell proliferation by HMDEs, we performed RNA-sequence analysis on CT-26 cells that were co-cultured with exosomes derived from normoxic macrophages (NMDEs) and HMDEs isolated from RAW264.7 cells. This analysis revealed significant changes in gene expression patterns. Specifically, 2745 genes were upregulated and 1412 genes were downregulated in CT-26 cells when exposed to HMDEs. The cutoff values used for this analysis were log2-fold changes ≤ -0.5 and ≥ 2.5 (**Figure 4A**). Gene ontology (GO) analysis pointed towards a significant role for these genes in promoting cell cycle transition, specifically in the G2/M phase (**Figure 4B**). Additionally, a total of 159 cell cycle-related genes were identified and subjected to analysis using the Kyoto Encyclopedia of Genes and Genomes (KEGG). This analysis revealed the involvement of the Hippo signaling pathway, FoxO signaling pathway, and Wnt signaling pathway in HMDE-mediated aberrant CT-26 cell growth (**Figure 4C**). While the Hippo signaling pathway is known to be associated with tumor growth and cell morphology, its role in HMDE-mediated cell proliferation has not been reported. Therefore, we further investigated the potential mechanism by which the Hippo signaling pathway functions in HMDE-mediated CT-26 cell proliferation. We analyzed the expression of target genes, *Ctgf* and *Cyr61*, in CT-26 cells treated with HMDEs to examine the activation of the Hippo signaling pathway. The results indicated the overexpression of these genes compared to the normoxic group (**Figures 4D**, **S6A, S6B, S6C**, and **S6D**), suggesting the activation of the Hippo signaling pathway in CT-26 cells. Furthermore, analysis using qPCR demonstrated that HMDEs regulate the aberrant activation of the Hippo signaling pathway in a non-transcriptional manner. This was supported by changes in the expression levels of key elements, including *Yap, Taz, Mst2, Lats1, Lats2, Tead, and 14-3-3* (**Figure 4E**). WB analysis showed increased levels of Yap and decreased levels of Lats1 in CT-26 cells co-cultured with HMDEs (**Figures 4F** and **S6E**). This indicates that HMDEs activate the Hippo signaling pathway through translational or posttranslational mechanisms. In the Hippo signaling pathway, Yap is typically inactivated by phosphorylation mediated by Lats1/2 kinases (12). We observed increased levels of active Yap and decreased levels of phosphorylated Yap at the S127 site in the HMDE group (**Figures 4G**, **S6F**, and **S6G**). This suggests that HMDEs regulate the abnormal activation of the Hippo signaling pathway at the posttranslational level. Notably, the nuclear accumulation of Yap is dependent on its dephosphorylation, which is a crucial post-transcriptional modification (22).

Enhanced nuclear accumulation of Yap was observed in the HMDEs group (**Figures 4H, I**), indicating the involvement of dephosphorylated Yap at S127 in HMDEs-mediated CT-26 cell proliferation. To further confirm the role of Yap degradation, we treated CT-26 cells co-cultured with NMDEs or HMDEs with cycloheximide (CHX), a protein synthesis inhibitor. The degradation rate of Yap was slower in the HMDEs group compared to the NMDE group. Yap degradation is known to occur through proteasomal degradation via ubiquitination following phosphorylation, which leads to a decrease in Yap levels (**Figures 4J** and **S8H**). Furthermore, we used the proteasome inhibitor MG132 to investigate whether Yap phosphorylation contributed to the degradation of Yap in the NMDE group. The results indicated that HMDEs increased Yap protein levels by inhibiting the proteasomal degradation of Yap (**Figure 4K**). To investigate the role of Hippo signaling pathway activation in promoting cell proliferation, we exposed CT-26 cells to verteporfin, a Yap inhibitor. Verteporfin inhibited HMDEs-mediated CT-26 cell proliferation, highlighting the essential role of the Hippo signaling pathway in HMDEs-mediated tumor growth (**Figure 4L**). Lats1 has been reported to be involved in Yap phosphorylation at the S127 site, mediated by MST1/2 or MAP4Ks in its hydrophobic motif (Thr1079 of Lats1). Interestingly, we observed suppressed phosphorylation of Lats1 at the Thr1079 site in CT-26 cells treated with HMDEs (**Figure 4G**). This suggests that the dephosphorylation of Lats1 may contribute to the abnormal activation of the Hippo signaling pathway mediated by HMDEs.

### 3.5 Exosomal Hsp90 protein contributed to tumor growth

**Figure 5:**
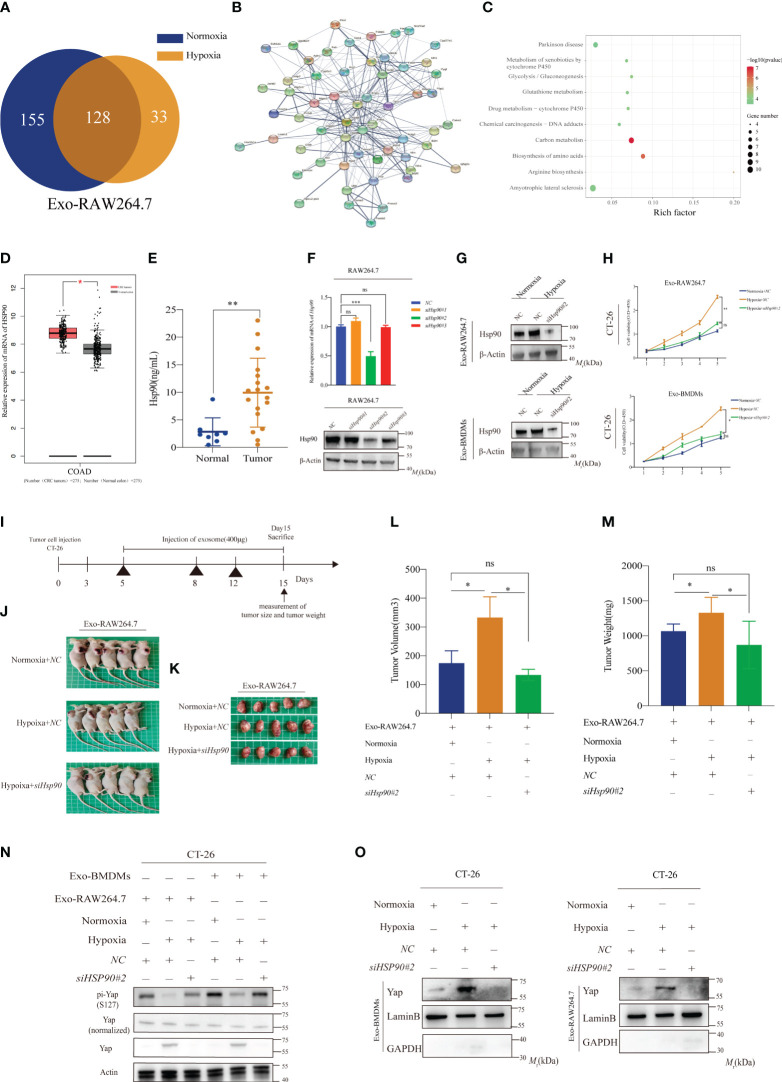
Exosomal Hsp90 protein contributes to tumor growth **(A)** MS analysis was performed to identify the differentially expressed proteins in exosomes from normoxic or hypoxic macrophages; **(B, C)** PPI and GO analyses according to the differentially expressed proteins in exosomes from normoxic or hypoxic macrophages; **(D)** Comparison of relative mRNA levels of FOXC1 in CRC and normal tissues. The data were downloaded from GEPIA (http://gepia.cancer-pku.cn/detail.php? gene = HSP90) (*P < 0.05); **(E)** The levels of Hsp90 protein were elevated in exosomes derived from the serum of CRC patients (**P < 0.01); **(F)** Transient knockdown of *Hsp90* gene expression by siRNA and expression efficiency was confirmed using WB, and β-actin was used as an internal control (ns, not significant, ***P < 0.001); **(G)** To confirm the expression in the exosomes from normoxic and hypoxic macrophages with or without Hsp90 knockdown; **(H)** The proliferation of CT-26 mediated by HMDEs was largely inhibited by *Hsp90* knockdown; (ns, not significant, *P < 0.05, **P < 0.01); **(I)** Schematic illustration showing the process of establishing a xenograft tumor model in BALB/c nude mice (400μg exosome per mouse); **(J, K)** Representative images showcasing tumor nodules in BALB/c nude mice treated with normoxic and hypoxic macrophages, with or without Hsp90 knockdown; **(L, M)** HMDEs-induced Hsp90 also has a large effect on tumor growth *in vivo*; (ns, not significant, *P < 0.05) **(N)** Decreased phosphorylation of Yap protein was observed in the HMDEs group with partial deletion of *Hsp90*; **(O)** Nuclear accumulation of Yap protein with partial deletion of Hsp90.

To investigate the role of Lats1 in regulating tumor growth and its potential connection with macrophage-derived exosomes under hypoxic conditions, we performed MS analysis to identify proteins present in these exosomes. This approach allowed us to investigate the role of proteins in macrophage-derived exosomes, which had received less attention in previous studies that primarily focused on noncoding RNAs (9). A total of 678 proteins were identified, with 293 proteins exclusively present in NMDEs and 193 proteins exclusively present in HMDEs. By calculating the enrichment ratio of protein abundance in HMDEs and NMDEs, we identified 68 proteins that exhibited significant differences between the two groups. Among these, 40 proteins were at least 2-fold enriched in HMDEs compared to NMDEs, while 28 proteins were more abundant in NMDEs (**Figure 5A** and **Supplementary 1**).

Through protein-protein interaction (PPI) and gene ontology (GO) analyses, we discovered that these proteins play a significant role in cell proliferation drivers, including glutathione metabolism, carbon metabolism, and arginine biosynthesis (**Figures 5B, C**). Of particular interest, we observed an upregulation of Hsp90 in HMDEs. Hsp90 is known to directly bind to Lats1 and activate the Hippo signaling pathway, especially during heat shock stress conditions (15). We aimed to investigate whether this effect extended to the proliferation of CT-26 cells mediated by macrophage exosomes under hypoxic conditions. Analyzing The Cancer Genome Atlas (TCGA) data, we observed that Hsp90 was upregulated in CRC tissues compared to normal tissues (**Figures 5D**). This observation was consistent with higher levels of Hsp90 protein in exosomes derived from the serum of CRC patients (**Figures 5E**). To investigate the role of Hsp90 in HMDEs-mediated cancer progression, we employed siRNA to silence Hsp90 in RAW264.7 cells. We selected siHsp90#2 for subsequent experiments (**Figure 5F**). As expected, HMDEs derived from Hsp90 knockdown cells exhibited decreased levels of Hsp90 (**Figures 5G** and **S7A**). Furthermore, the proliferation of CT-26 cells was downregulated after Hsp90 knockdown (**Figure 5H**), and similar results were observed *in vivo* (**Figures 5I–M**). To further confirm the effects of Hsp90 on promoting Hippo signaling pathway activation, we conducted a WB assay to examine Yap expression in CT-26 cells treated with HMDEs, with or without Hsp90 knockdown. We observed increased Yap phosphorylation at the S127 site and decreased Yap expression in the Hsp90 knockdown group, indicating that HMDEs promoted the activation of the Hippo signaling pathway in an Hsp90-dependent manner (**Figure 5N**). Additionally, the nuclear accumulation of Yap was decreased in the Hsp90 knockdown group (**Figure 5O**). These findings collectively demonstrated that elevated levels of Hsp90 in HMDEs were responsible for HMDEs-mediated tumor growth both *in vitro* and *in vivo* by promoting the activation of the Hippo signaling pathway.

### 3.6 Elevated Hsp90 in HMDEs regulates Yap dephosphorylation and nuclear accumulation through directly binding to Lats1

**Figure 6:**
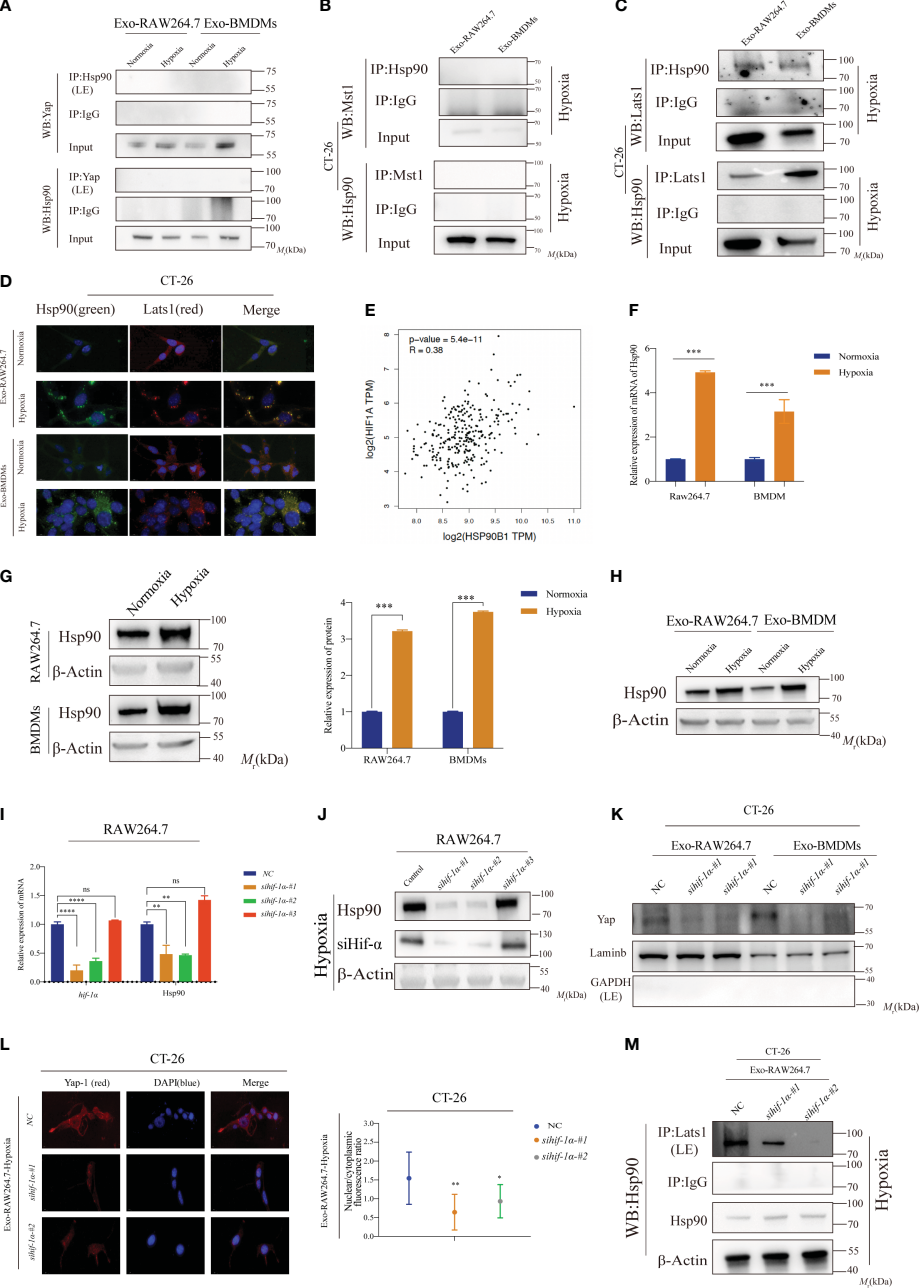
Elevated Hsp90 in HMDEs regulates Yap dephosphorylation and nuclear accumulation through directly binding to Lats1 **(A, B)** CoIP assay was conducted to confirm whether Yap or Mst1 bound to Hsp90 in CT-26 cells mediated by NMDEs or HMDEs; **(C, D)** Lats1 protein directly bound to Hsp90 protein in CT-26 cells mediated by HMDEs; **(E)** The data from TCGA database indicated that HIF-1α might contribute to HSP90 expression; **(F, G)** Both qPCR and WB assays were employed to rigorously confirm the upregulation of Hsp90 expression under hypoxic conditions; **(H)** Hypoxia induced an increase in the expression of Hsp90 protein in both RAW264.7 cells and exosomes derived from BMDMs; **(I, J)** *Hif-1α* knockdown decreased Hsp90 expression at both the transcriptional and translational levels; (^∗^P < 0.05, ^∗∗^P < 0.01, ^***^P < 0.001, ^****^P < 0.0001) **(K)** Decreased nuclear accumulation of Yap protein was found in the WB assay; (^∗^P < 0.05, ^∗∗^P < 0.01, ^***^P < 0.001, ^****^P < 0.0001) **(L)** Partial downregulation of *HIF-1α* expression led to a reduction in the nuclear accumulation of Yap protein; **(M)** Co-IP assay showed that *Hif-1α* knockdown pulled down less Lats1 protein. ns, not significant.

To further investigate the intricate mechanism by which Hsp90 modulates the activation of the Hippo signaling pathway, the co-IP assay was performed to examine the potential direct interaction between Hsp90 and Yap. Surprisingly, no direct interaction between Hsp90 and Yap was observed in our study (**Figure 6A**). Given Lats1 seemed to play a role in HMDEs-mediated CT-26 cell proliferation, as evidenced by increased dephosphorylation at Thr1079 (**Figure 4G**). Therefore, we next explored the potential interaction between Hsp90 and Mst1. Mst1 is a significant kinase responsible for phosphorylating Lats1 upon activation by MAP4Ks (15). However, the results of co-IP assay indicated that there was no detectable interaction between Hsp90 and Mst1 (**Figure 6B**). Remarkably, a direct binding between Hsp90 and Lats1 was discovered (**Figures 6C, D**). This discovery implied that Hsp90 has the potential to impede the Yap-dependent phosphorylation of Lats1 by forming a physical bond with Lats1, consequently facilitating the activation of the Hippo signaling pathway. To elucidate the underlying mechanism responsible for the upregulation of Hsp90 in hypoxic environments, we next explored the potential regulatory role of HIF-1α. HIF-1α is recognized for its ability to react to hypoxic stress and stimulate the transcription of downstream target genes. By examining the mRNA expression levels of HIF-1α and HSP90 in TCGA dataset, a potential correlation between these two genes was observed (**Figure 6E**). To establish the regulatory association between HIF-1α and HSP90, qPCR and WB analyses were performed. The results demonstrated that HIF-1α played a role in the regulation of HSP90 expression both at the transcriptional and translational levels (**Figures 6F, G**). Additionally, elevated levels of Hsp90 protein were observed in HMDEs in comparison to NMDEs (**Figure 6H**). Knockdown of Hif-1α led to a reduction in Hsp90 protein levels (**Figures 6I, J**) The viability of CT-26 cells significantly decreased after they were treated with supernatant-derived hypoxic macrophages that had undergone partial *Hif-1α* knockdown (**Figures S8A, S8B**). These findings collectively suggested that Hsp90 might be a direct target gene of *HIF-1α* and that HIF-1α could facilitate the activation of the Hippo signaling pathway during HMDEs-mediated CT-26 cell proliferation by promoting the upregulation of Hsp90. To ascertain the involvement of HIF-1α in the abnormal activation of the Hippo signaling pathway in CT-26 cells co-cultured with HMDEs, an investigation was conducted to assess the nuclear translocation of Yap in these cells. Partial downregulation of *HIF-1α* expression led to a reduction in the nuclear accumulation of Yap protein (**Figures 6K, L**). Additionally, the affinity between Lats1 and Hsp90 was diminished upon partial knockdown of HIF-1α (**Figure 6M**). Overall, the results of this study indicated that the activation of the Hippo signaling pathway during HMDEs-mediated CT-26 cell proliferation was mediated by HIF-1α, which was achieved through the overexpression of Hsp90.

### 3.7 *Hsp90* is the target gene of Hif-1α

**Figure 7:**
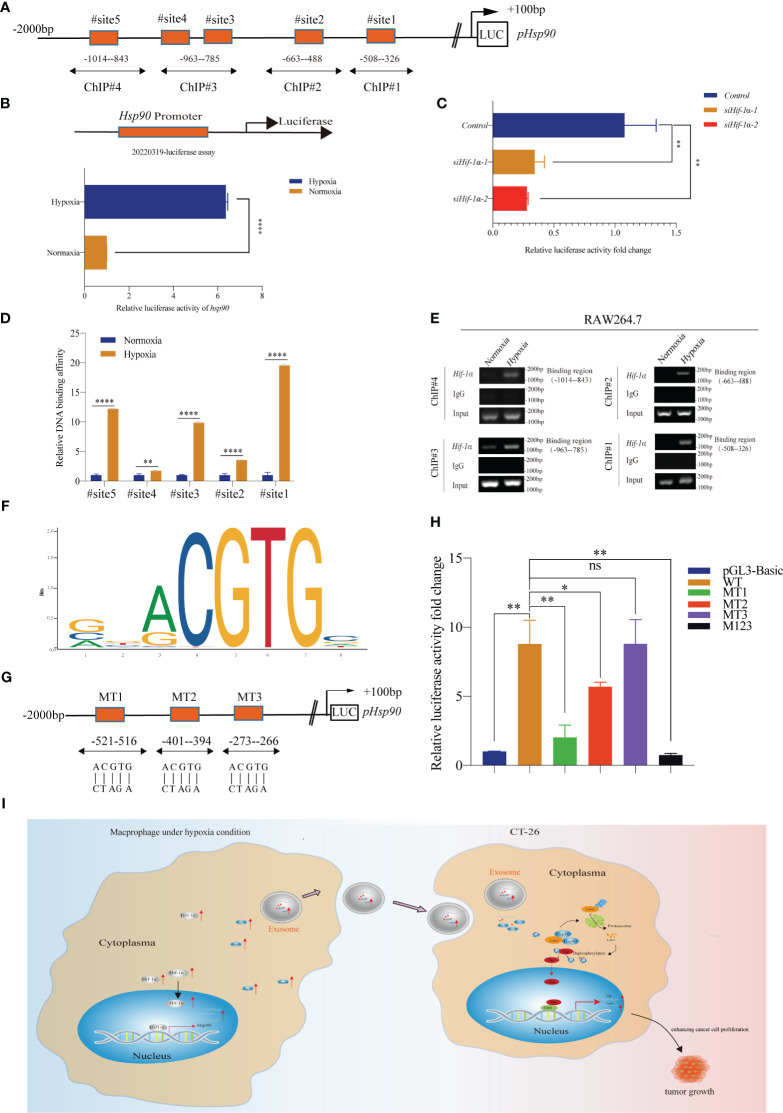
*Hsp90* is the target gene of Hif-1α **(A)** Schematic diagram showing the potential binding region for *Hif-1α*; **(B, C)** A luciferase reporter assay was conducted to verify that hypoxia regulated the expression of Hsp90 through Hif-1α at the transcriptional level; (**P < 0.01, ****P < 0.0001); **(D, E)** ChIP assay was used to measure the potential Hif-1α binding sites at the Hsp90 promoter (^∗∗^P < 0.01, ^****^P < 0.0001); **(F)** Schematic diagram showing that YYYCGTGY might be the special motif of the *Hif-1α* protein; **(G)** Luciferase reporter assay of three *Hsp90* promoter region mutations in the RAW264.7 cells; **(H)** Luciferase reporter results indicated that Hif-1α directly bound to the predicted sites (-521– -516 bp and -401– -391 bp) of the *Hsp90* promoter (ns, not significant, *P < 0.05, **P < 0.01); **(I)** Proposed mechanism by which HMDEs regulated the tumor growth of CT-26 cells.

To examine the potential targeting of Hsp90 by Hif-1α, a luciferase reporter plasmid (pGL3-Hsp90-Luc) was constructed, incorporating the sequence spanning from +100 bp to -2000 bp of the promoter region of the murine Hsp90 gene (**Figure 7A**). Under hypoxic conditions, upregulation of *Hsp90* expression was observed in RAW264.7 cells (**Figure 7B**). Significantly, the knockdown of *Hif-1α* resulted in a notable inhibition of Hsp90 expression (**Figure 7C**), suggesting that Hif-1α plays a role in the transcriptional upregulation of *Hsp90*. Subsequently, a ChIP assay was performed to investigate the potential binding sites of Hif-1α within the promoter region of *Hsp90*. We have successfully identified four potential binding sites of Hif-1α in the promoter region of *Hsp90* (**Figures 7D, E**). This finding strongly supports the hypothesis that *Hsp90* is a direct target gene of Hif-1α. Subsequently, a JASPAR analysis was conducted to ascertain the potential DNA-binding sequence for Hif-1α. The analysis revealed the presence of the motif YYYACGTGY (**Figure 7F**). Based on the analysis results from JASPAR, three potential binding sites for Hif-1α were identified in the promoter region of the Hsp90 gene (**Figure 7G**). To confirm the precise binding sites, we implemented mutations within these binding sites, altering the sequence ACGTG to CTAGA (**Figure 7G**). Luciferase reporter assays were performed to assess the impact of mutated binding sites on luciferase activity. The results demonstrated a significant decrease in luciferase activity in the MT1, MT2, and MT123 groups, indicating impaired binding. However, no significant difference in luciferase activity was observed in the MT3 group (**Figure 7H**). The findings of this study provide compelling evidence that Hif-1α exhibits specific interactions with the -521− -516 bp and -401− -394 bp regions of the *Hsp90* promoter, resulting in the increased expression of Hsp90. Consequently, the upregulation of Hsp90 protein in HMDEs led to increased levels of this protein, which played a critical role in activating the Hippo signaling pathway and the promotion of CRC cell proliferation.

## 4 Discussion

Macrophages, which are a type of immune cell, are present within the tumor microenvironment (6). Tumor cells are subject to interaction with these entities, which subsequently impact the immune response directed toward the tumor. In the context of the tumor microenvironment, macrophages have the ability to undergo polarization into two distinct phenotypes known as M1 and M2 (23). M1 macrophages exhibit an anti-tumor role by releasing pro-inflammatory cytokines, including interleukin-12 (IL-12) and tumor necrosis factor-alpha (TNF-α). They additionally facilitate the presentation of antigens to stimulate the activation of cytotoxic T cells, thereby promoting an immune response directed against the tumor. On the contrary, M2 macrophages exhibit pro-tumor characteristics and are closely linked to the advancement of tumors. They produce anti-inflammatory cytokines, including interleukin-10 (IL-10) and TGF-β, which play a role in promoting tumor cell survival, angiogenesis, and immunosuppression (24). M2 macrophages play a crucial role in promoting tumor invasion and metastasis through their ability to remodel the extracellular matrix. Tumor cells have the ability to actively manipulate the polarization of macrophages in order to gain an advantage. They secrete molecules such as colony-stimulating factor-1 and chemokines, which serve to recruit macrophages and facilitate their polarization towards the M2 phenotype. In turn, M2 macrophages contribute to the facilitation of tumor growth by generating growth factors and facilitating angiogenesis. Hypoxia, a characteristic feature of solid tumors, induces the polarization of macrophages towards the M2 phenotype, which is recognized for its role in facilitating tumor growth and progression (25). Furthermore, a growing body of evidence has revealed that hypoxia plays a crucial role in the increased secretion of exosomes derived from tumor cells. This phenomenon promotes communication between tumor cells and macrophages, thereby facilitating tumor progression (26–28). However, the impact of hypoxia on the release of exosomes from macrophages, as well as the involvement of hypoxia-induced microenvironmental changes in the growth and progression of CRC and the underlying mechanisms, have yet to be fully understood. In the present study, our objective was to examine the role of HMDEs in the promotion and advancement of CRC. Our findings indicate that when exposed to hypoxic conditions, macrophages exhibit an increased release of exosomes. These HMDEs have been shown to play a crucial role in facilitating the growth of CRC cells. Specifically, they promote cell cycle progression and suppress cell apoptosis, thereby contributing to the overall proliferation of CRC cells. The aforementioned findings indicate that HMDEs play a role as a carcinogenic factor in the progression of CRC.

Additionally, we investigated the molecular mechanisms underlying these effects. We observed that the condition of hypoxia resulted in an upregulation of Hif-1α expression. This upregulated Hif-1α directly interacted with specific regions (-521− -516 bp and -401− -394 bp regions) of the Hsp90 promoter. Consequently, this interaction led to an increased expression of Hsp90 and elevated levels of Hsp90 protein within HMDEs. The interaction between Hsp90 and large tumor suppressor kinase 1 (Lats1), a crucial element of the Hippo signaling pathway, led to the deactivation of Lats1 and the inhibition of Yap phosphorylation. Consequently, the series of events led to the ultimate inactivation of the Hippo signaling pathway. Notably, our comprehensive proteomic analysis revealed that HMDEs exhibited enrichment of 678 distinct proteins. Among these proteins, 40 displayed elevated levels, while 28 exhibited decreased levels in comparison to NMDEs (**Supplementary 1**). These findings exhibited disparities when compared to the results reported in studies investigating exosomes derived from tumor cells under hypoxic conditions (8, 29, 30).

The observed variations can be attributed to the utilization of different cell types and the specific hypoxic conditions employed. Additionally, our study has shown that HMDEs play a significant role in the progression of CRC through the regulation of the Hippo signaling pathway. This finding is consistent with previous research that has highlighted the promoting effect of M2-like macrophages on tumor progression. The Hippo signaling pathway plays a pivotal role in regulating a wide range of biological processes, such as cell growth, apoptosis, and control of organ size. In the aforementioned signaling cascade, MST1/2 kinases phosphorylate LATS1/2 kinases, and the phosphorylated LATS1/2 kinases subsequently phosphorylate YAP. Phosphorylated YAP interacts with 14-3-3, leading to the sequestration of YAP in the cytoplasm and inhibiting its translocation to the nucleus (31). When the Hippo pathway is rendered inactive, the YAP undergoes dephosphorylation, leading to its accumulation in the nucleus. Subsequently, YAP forms complexes with TEAD, thereby facilitating the transcription of target genes. This molecular mechanism promotes tumor progression in a variety of cancers, including CRC (14). Previous research has provided evidence of the impact of macrophage-derived exosomes on tumor advancement and the control of YAP nuclear translocation. However, these investigations have primarily focused on normoxic conditions, leaving the effects of HMDEs and the underlying mechanisms unexplored (8, 28, 32). Our study findings indicated that the use of HMDEs resulted in the inhibition of YAP phosphorylation, facilitated YAP nuclear translocation, and played a role in the progression of CRC.

HSP90, a constituent of the heat shock protein family, functions as an ATP-dependent molecular chaperone (33). The stabilization and activation of client proteins, which are crucial for cell survival and tumor growth, play a significant role in tumor groth (34, 35). HSP90 plays a crucial role in various cellular processes, including proliferation, viability, protein folding, and degradation (32, 33). The upregulation of HSP90 facilitates the survival of tumor cells in challenging microenvironments, such as hypoxia, thereby promoting the persistence of mutations that contribute to the development of malignancy (36). HSP90 has been implicated in the inactivation of the Hippo signaling pathway through the inhibition of Lats1 phosphorylation under conditions of heat stress (15). The HSP90 inhibitor 17-AAG has demonstrated inhibitory effects on cell proliferation through the modulation of the Hippo signaling pathway. In this study, we examined the impact of exosomal Hsp90 derived from macrophages on the progression of CRC. We discovered a direct interaction between Hsp90 and Lats1, resulting in the dephosphorylation of Yap, its translocation into the nucleus, and subsequent proliferation of CRC cells. Additionally, an investigation was conducted to examine the correlation between hypoxia and the expression of HSP90. HSP90 expression was observed to exhibit a positive correlation with the expression of HIF-1α protein in tissues of hepatocellular carcinoma. In our study, we have provided evidence to support the notion that the exposure of macrophages to hypoxic conditions leads to an upregulation of HIF-1α expression. This, in turn, results]ed in an increased expression of HSP90 through direct binding to specific regions of the *Hsp90* promoter (**Figure 7I**).

In summary, our study elucidated a substantial contribution of hypoxic macrophage-derived exosomal Hsp90 in the CRC progression through its interaction with the Hippo signaling pathway. These findings provided valuable insights into the intricate interaction between macrophages and CRC cells in hypoxic environments.

## Data availability statement

The original contributions presented in the study are publicly available. This data can be found here: https://bigd.big.ac.cn/gsa/browse/CRA013204.

## Ethics statement

The studies involving humans were approved and the studies were conducted in accordance with the approved guidelines and regulations. Prior to participation, written informed consent was obtained from all individual participants or their legal guardians, ensuring their understanding of the study’s purpose, procedures, potential risks, and benefits. The study was assigned the Clinical Ethics Trial No (315). Aiguo Lu, Ph.D. The studies were conducted in accordance with the local legislation and institutional requirements. The participants provided their written informed consent to participate in this study. The animal study was approved by The experimental protocol adhered to the ethical principles outlined in the Declaration of Helsinki and received approval from the Human Ethics Committee of the Department of General Surgery, Ruijin Hospital, Shanghai Jiaotong University School of Medicine (Shanghai, China). The study was conducted in accordance with the local legislation and institutional requirements.

## Author contributions

HZ: Conceptualization, Funding acquisition, Methodology, Resources, Writing – original draft, Writing – review & editing. JJ: Conceptualization, Data curation, Methodology, Project administration, Writing – original draft, Writing – review & editing. WW: Methodology, Project administration, Writing – review & editing. LZ: Funding acquisition, Methodology, Project administration, Resources, Writing – review & editing. BS: Methodology, Project administration, Writing – review & editing. YC: Investigation, Resources, Writing – review & editing. YX: Methodology, Resources, Writing – review & editing. WF: Methodology, Resources, Writing – review & editing. WY: Methodology, Project administration, Resources, Writing – review & editing. AL: Methodology, Project administration, Resources, Writing – review & editing.

## Supplementary material

SUPPLEMENTARY FIGURE S1 The activation of HIF-1α of RAW264.7 and BMDMs under hypoxic conditions. **(A)** qPCR assay was performed to confirm the activation of HIF-1α after subjecting these cell lines (RAW264.7 and BMDMs) to hypoxic conditions.

SUPPLEMENTARY FIGURE S2 Quantitative analysis of WB resulted in the exosomal marker under the hypoxic conditions and normoxic conditions. **(A, B)** WB results have undergone quantitative analysis, confirming elevated levels of exosomal proteins, including Alix, Annexin, Flotillin-1, and Hsp70, in the hypoxic group.

SUPPLEMENTARY FIGURE S3 GW4869 inhibited exosome secretion both under hypoxic conditions and normoxic conditions. **(A)** WB experiments and quantitative analysis confirmed the inhibitory effect of GW4869 on macrophage exosome secretion both under hypoxic conditions and normoxic conditions.

SUPPLEMENTARY FIGURE S4 Cell viability of CT-26 and MC-38 cells following treatment with elevated exosome doses. **(A, B)** The CCK-8 assay confirmed that the viability of CT-26 and MC-38 cells increased with higher exosome dosages.

SUPPLEMENTARY FIGURE S5 Inhibition of exosome secretion decreased in cell cycle transition and cell apoptosis. **(A, B)** The suppression of exosome release from RAW264.7 or BMDMs through the use of GW4869 resulted in a reduction in cell cycle transition and apoptosis in CT-26 cells.

SUPPLEMENTARY FIGURE S6 HMDEs play a role in promoting tumor growth by inhibiting the Hippo signaling pathway. **(A)** Quantitative analysis of the WB assay indicated increased expression levels of CTGF and CYR61 in CT-26 cells treated with HMDEs or NMDEs. **(B)** qPCR assay was performed to confirm the expression of CTGF and CYR61 at the transcriptional level in MC-38 cells treated with HMDEs or NMDEs. **(C, D)** WB assay and quantitative analysis were performed to confirm the expression of CTGF and CYR61 at the translational level in MC-38 cells treated with HMDEs or NMDEs. **(E)** Quantitative analysis of WB showed the expression of hippo-related proteins in CT-26 cells treated with HMDEs or NMDEs. **(F, G)** Quantitative analysis of WB revealed the increased pLats1, pYap, and active Yap in CT-26 cells or MC-38 cells treated with HMDEs or NMDEs. **(H)** Quantitative analysis of WB leads to a decrease in Yap levels in CT-26 cells treated with HMDEs or NMDEs.

SUPPLEMENTARY FIGURE S7 Hsp90 levels were decreased in Hsp90 knockdown cells HMDEs. **(A)** The quantitative analysis of the WB revealed that exosomes derived from Hsp90 knockdown exhibited reduced levels of Hsp90 in the HMDEs.

SUPPLEMENTARY FIGURE S8 Partial knockdown of *Hif-1α* altered the viability of CT-26 cells. **(A)** The viability of CT-26 cells significantly decreased after they were treated with supernatant-derived hypoxic macrophages that had undergone partial *Hif-1α* knockdown.
