# Supplementary figures and images for: Unveiling the role of hypoxic macrophage-derived exosomes in driving colorectal cancer progression
Source: Front Immunol. 2023 Nov 9;14:1260638. doi: 10.3389/fimmu.2023.1260638 (PMC10666760; doi:10.3389/fimmu.2023.1260638)

A

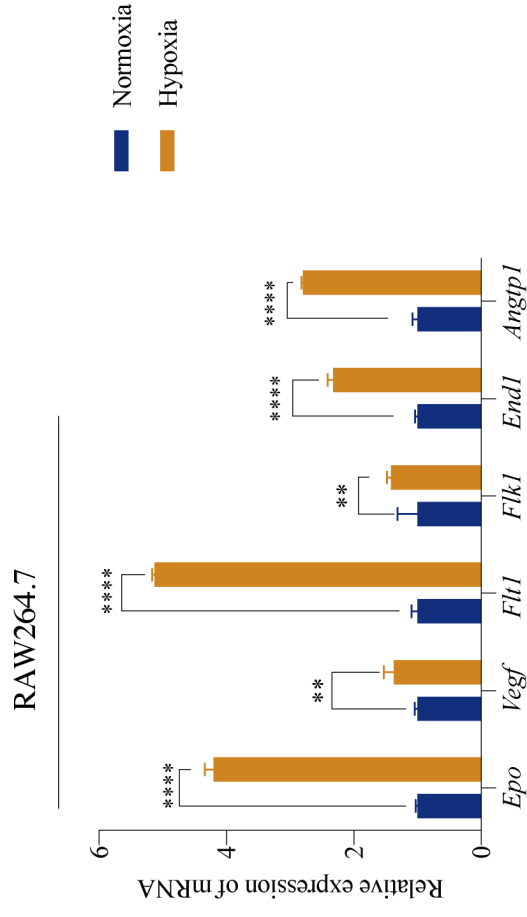

B

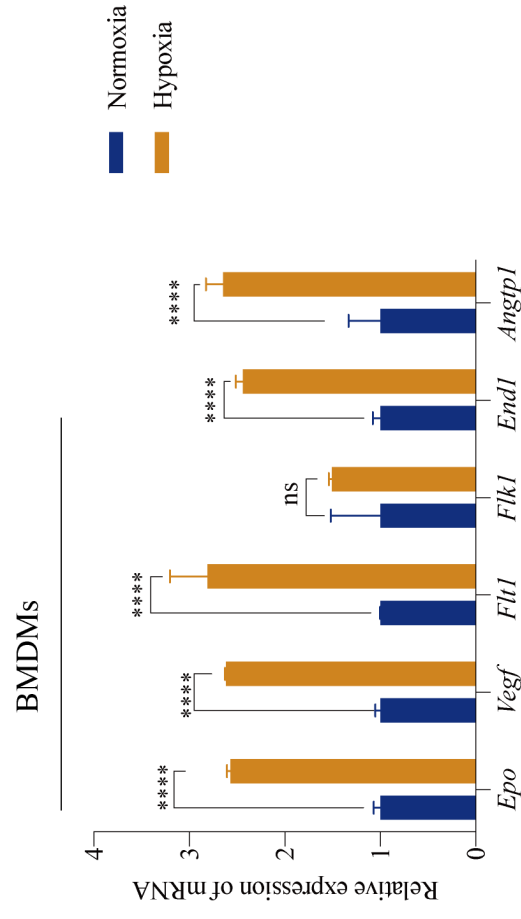

Supplement: SUPPLEMENTARY FIGURE S1 — The activation of HIF-1α of RAW264.7 and BMDMs under hypoxic conditions. (A) qPCR assay was performed to confirm the activation of HIF-1α after subjecting these cell lines (RAW264.7 and BMDMs) to hypoxic conditions. [file Image_1.pdf]

A

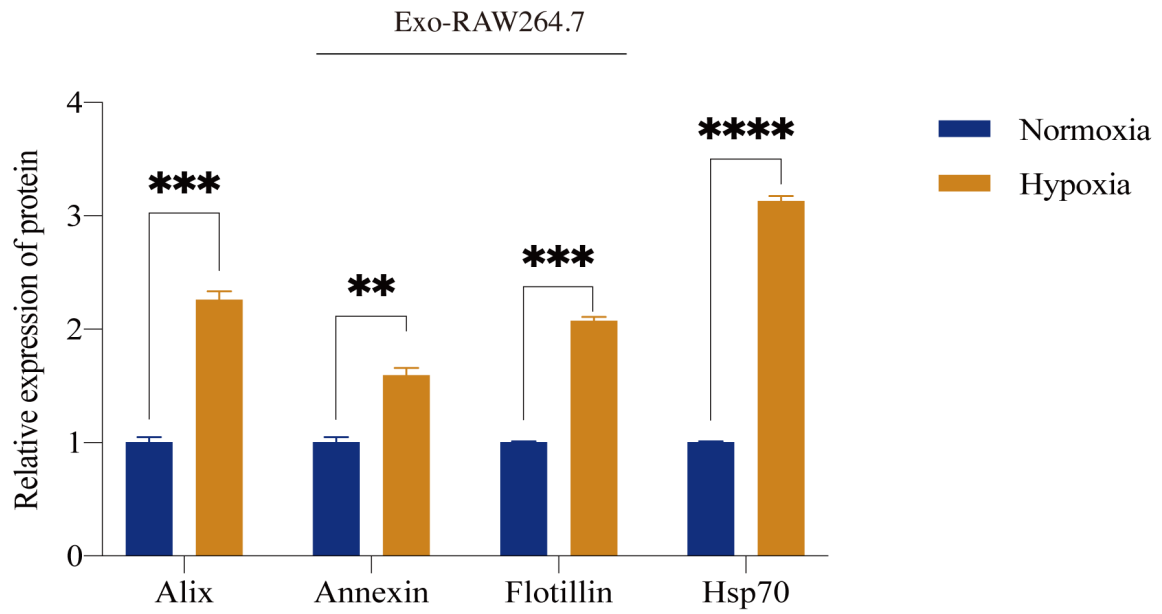

B

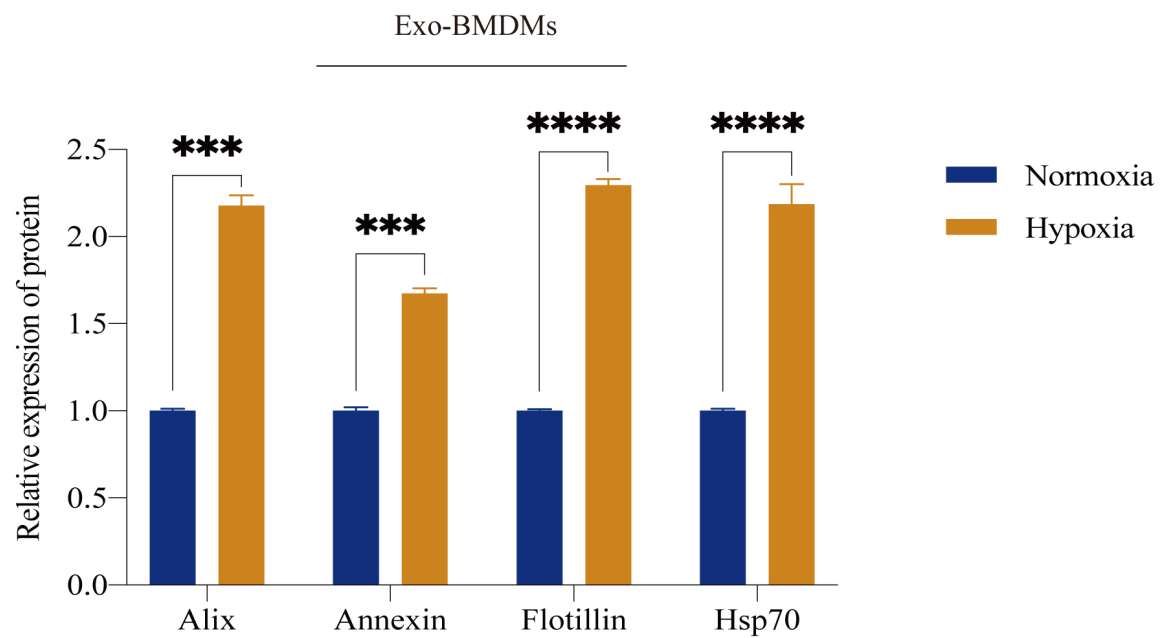

Supplement: SUPPLEMENTARY FIGURE S2 — Quantitative analysis of WB resulted in the exosomal marker under the hypoxic conditions and normoxic conditions. (A, B) WB results have undergone quantitative analysis, confirming elevated levels of exosomal proteins, including Alix, Annexin, Flotillin-1, and Hsp70, in the hypoxic group. [file Image_2.pdf]

Exo-RAW264.7

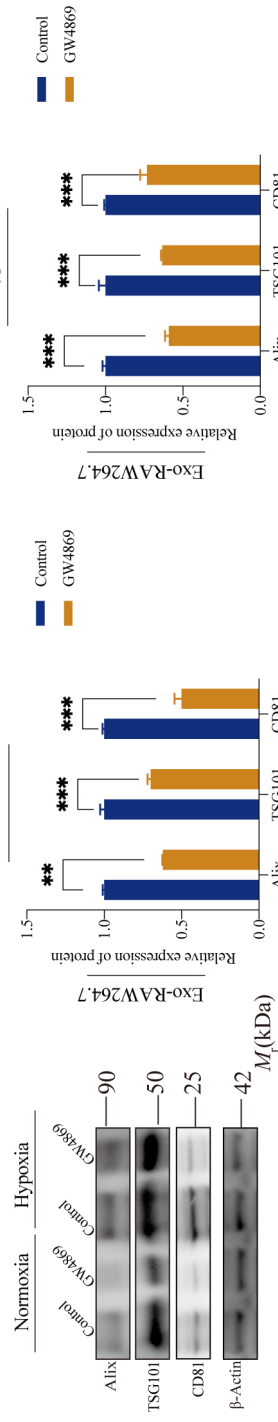

Supplement: SUPPLEMENTARY FIGURE S3 — GW4869 inhibited exosome secretion both under hypoxic conditions and normoxic conditions. (A) WB experiments and quantitative analysis confirmed the inhibitory effect of GW4869 on macrophage exosome secretion both under hypoxic conditions and normoxic conditions. [file Image_3.pdf]

A

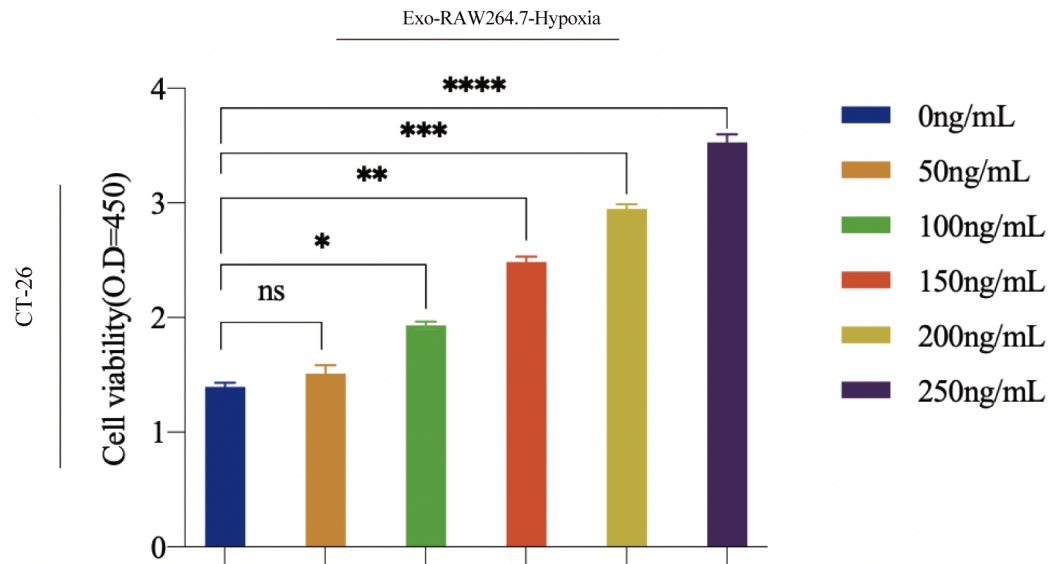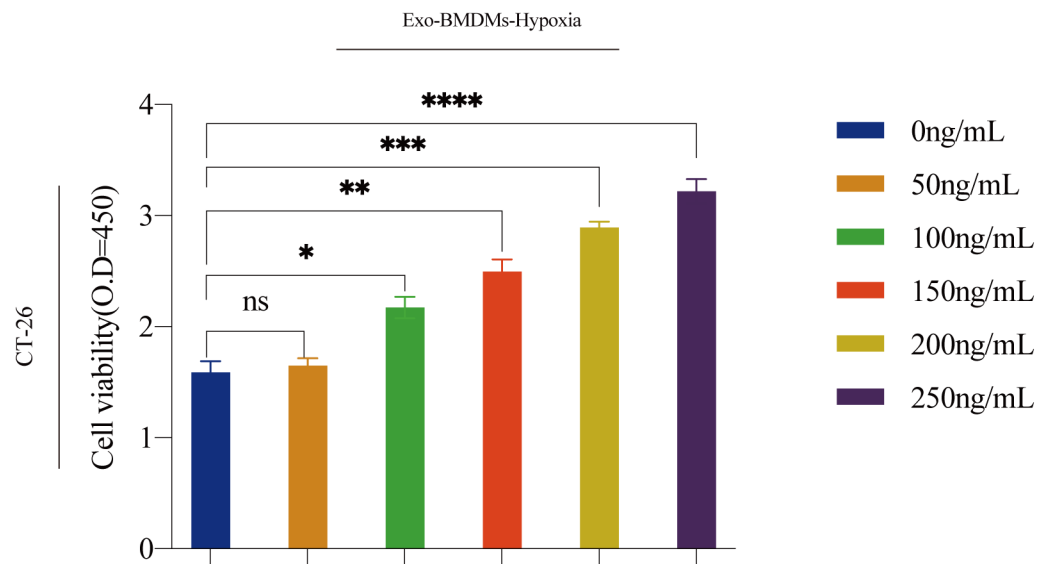

Supplement: SUPPLEMENTARY FIGURE S4 — Cell viability of CT-26 and MC-38 cells following treatment with elevated exosome doses. (A, B) The CCK-8 assay confirmed that the viability of CT-26 and MC-38 cells increased with higher exosome dosages. [file Image_4.pdf]

A

CT-26

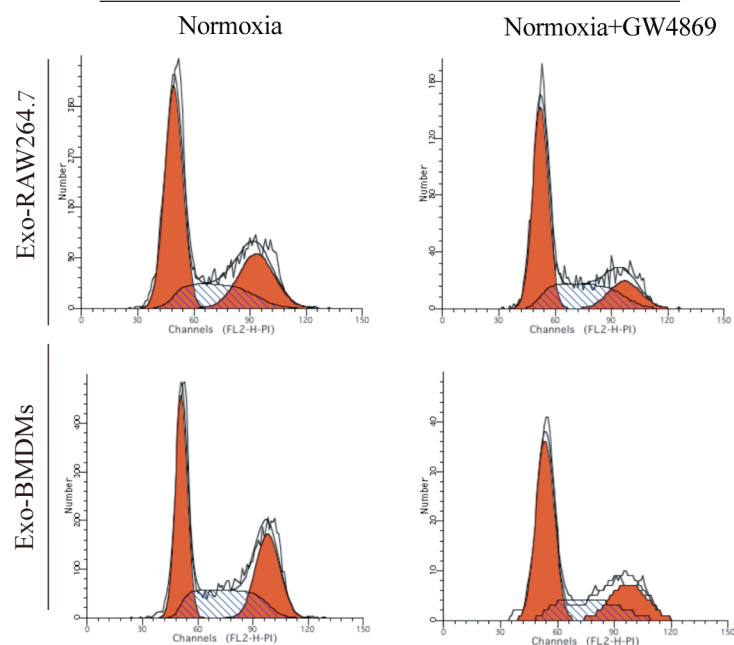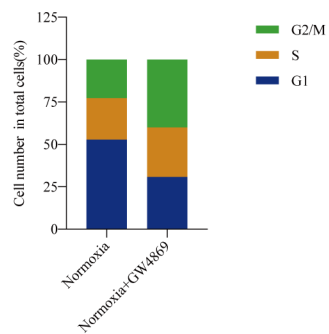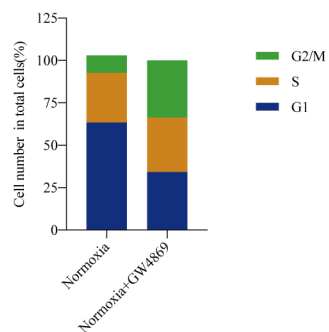

B

CT-26

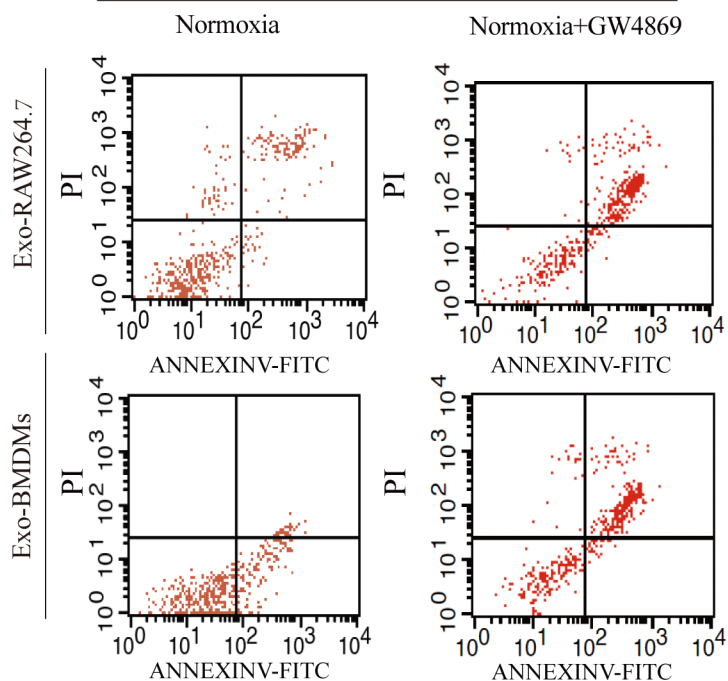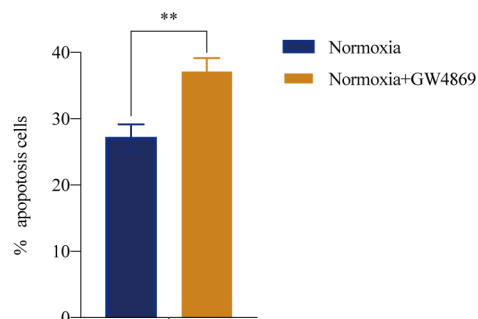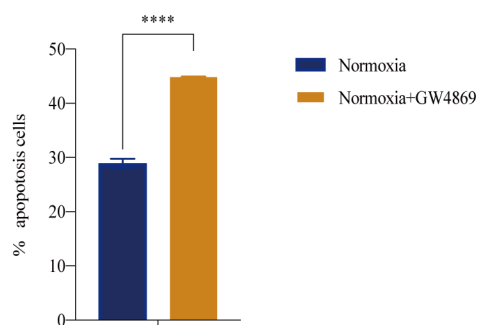

Supplement: SUPPLEMENTARY FIGURE S5 — Inhibition of exosome secretion decreased in cell cycle transition and cell apoptosis. (A, B) The suppression of exosome release from RAW264.7 or BMDMs through the use of GW4869 resulted in a reduction in cell cycle transition and apoptosis in CT-26 cells. [file Image_5.pdf]

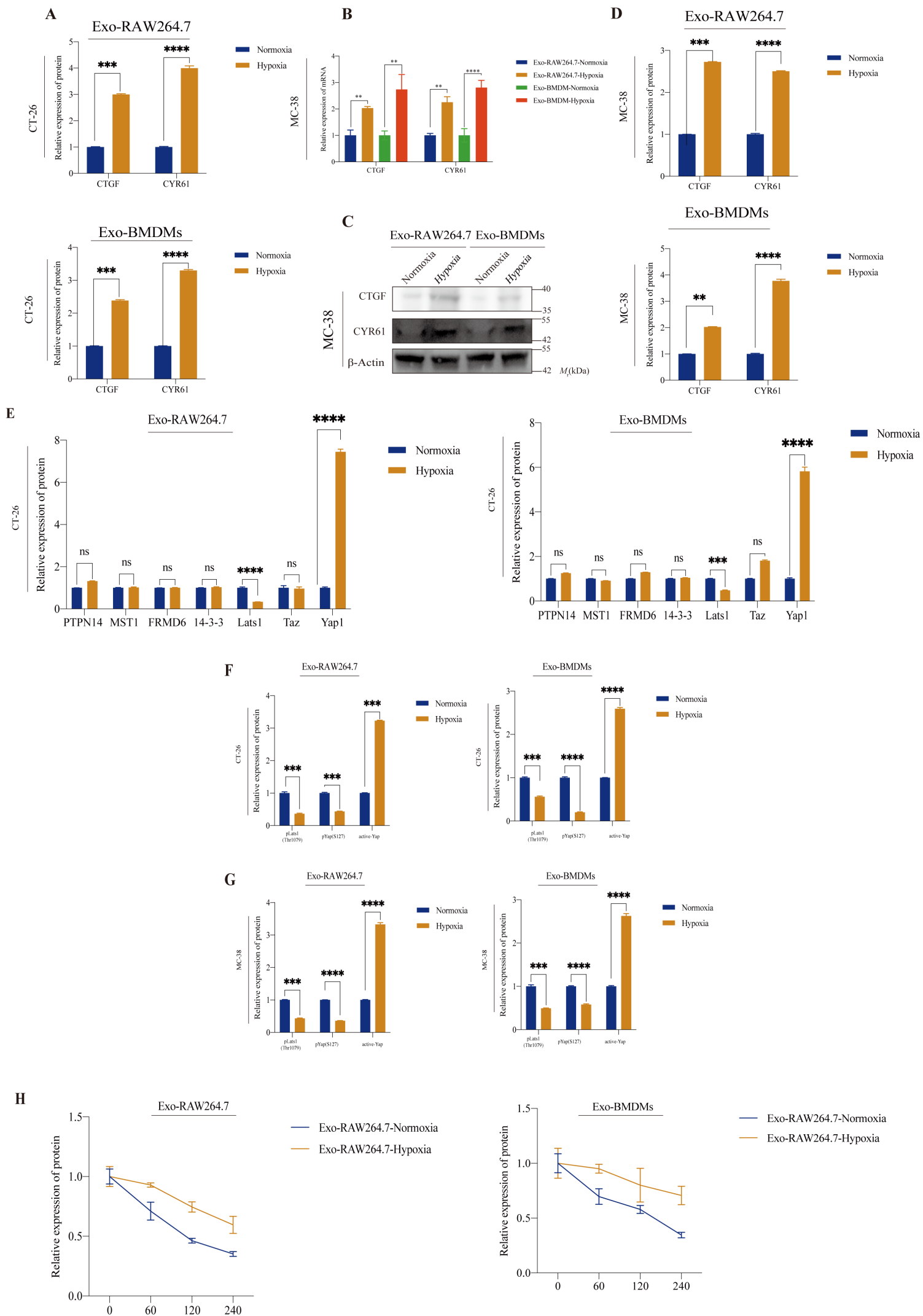

Supplement: SUPPLEMENTARY FIGURE S6 — HMDEs play a role in promoting tumor growth by inhibiting the Hippo signaling pathway. (A) Quantitative analysis of the WB assay indicated increased expression levels of CTGF and CYR61 in CT-26 cells treated with HMDEs or NMDEs. (B) qPCR assay was performed to confirm the expression of CTGF and CYR61 at the transcriptional level in MC-38 cells treated with HMDEs or NMDEs. (C, D) WB assay and quantitative analysis were performed to confirm the expression of CTGF and CYR61 at the translational level in MC-38 cells treated with HMDEs or NMDEs. (E) Quantitative analysis of WB showed the expression of hippo-related proteins in CT-26 cells treated with HMDEs or NMDEs. (F, G) Quantitative analysis of WB revealed the increased pLats1, pYap, and active Yap in CT-26 cells or MC-38 cells treated with HMDEs or NMDEs. (H) Quantitative analysis of WB leads to a decrease in Yap levels in CT-26 cells treated with HMDEs or NMDEs. [file Image_6.pdf]

**A**

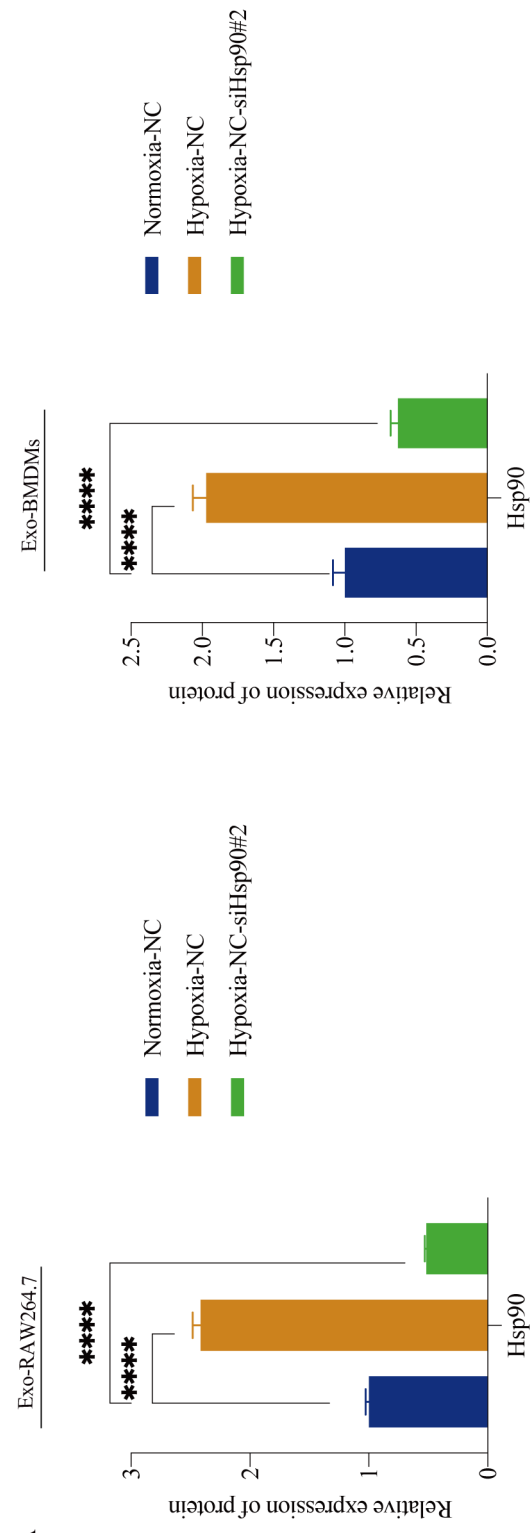

Supplement: SUPPLEMENTARY FIGURE S7 — Hsp90 levels were decreased in Hsp90 knockdown cells HMDEs. (A) The quantitative analysis of the WB revealed that exosomes derived from Hsp90 knockdown exhibited reduced levels of Hsp90 in the HMDEs. [file Image_7.pdf]
