# Supplementary material for: Unveiling the role of hypoxic macrophage-derived exosomes in driving colorectal cancer progression
Source: Front Immunol. 2023 Nov 9;14:1260638. doi: 10.3389/fimmu.2023.1260638 (PMC10666760; doi:10.3389/fimmu.2023.1260638)
Supplement: SUPPLEMENTARY FIGURE S8 — Partial knockdown of Hif-1α altered the viability of CT-26 cells. (A) The viability of CT-26 cells significantly decreased after they were treated with supernatant-derived hypoxic macrophages that had undergone partial Hif-1α knockdown. [file Image_8.pdf]

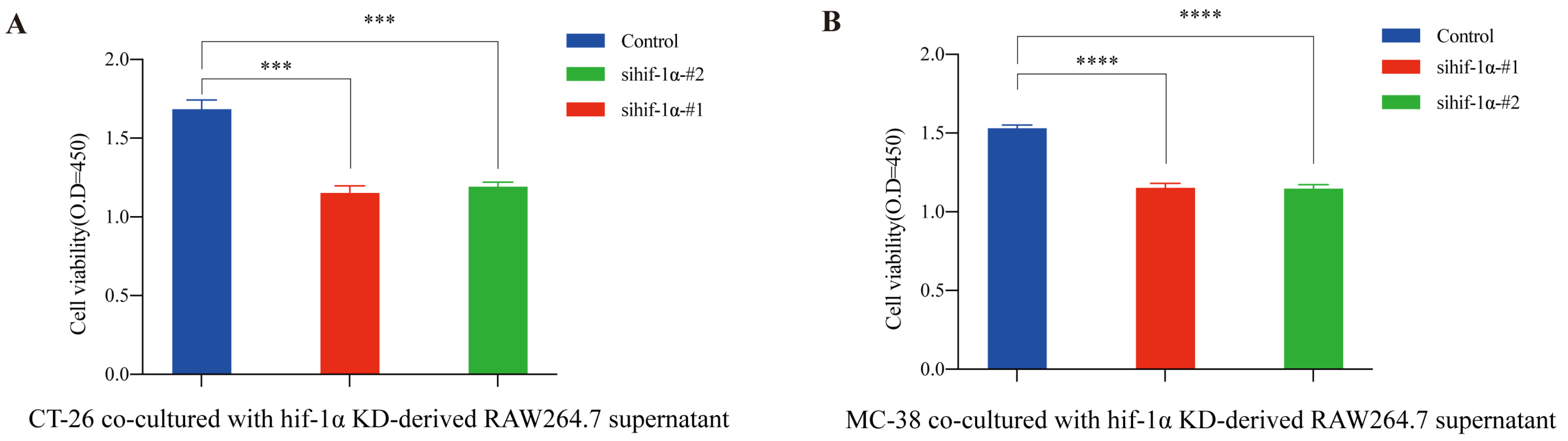
A
***
***
Control
sihif-1α-#2
sihif-1α-#1
Cell viability(O.D=450)
2.0
1.5
1.0
0.5
0.0
CT-26 co-cultured with hif-1α KD-derived RAW264.7 supernatant
B
****
****
Control
sihif-1α-#1
sihif-1α-#2
Cell viability(O.D=450)
2.0
1.5
1.0
0.5
0.0
MC-38 co-cultured with hif-1α KD-derived RAW264.7 supernatant
